# Optimized frequency stabilization in hybrid renewable power grids with integrated energy storage systems using a modified fuzzy-TID controller

**DOI:** 10.1038/s41598-025-02011-0

**Published:** 2025-06-20

**Authors:** Mohamed Khamies, Ahmed H. A. Elkasem, Salah Kamel, Mohamed Hashem

**Affiliations:** 1 Department of Electrical Engineering, Faculty of Engineering, Sohag University, Sohag, 82524 Egypt; 2 Department of Electronics and Communications, Faculty of Engineering, Sphinx University, Assiut , Egypt; 3 Electrical Engineering Department, Faculty of Engineering, Aswan University, Aswan, 81542 Egypt; 4 Holding Company for Water and Wastewater, Sohag, Egypt

**Keywords:** Frequency stability, Fuzzy based integral–tilt-derivative, Energy storage systems, Unified power flow controller, Renewable energy sources, Optimization, Sea horse optimizer, Electrical and electronic engineering, Energy grids and networks

## Abstract

This article presents several innovative methods to mitigate frequency deviations in hybrid renewable power grids (HRPGs) with high penetration of renewable energy sources (RESs). Two models of the HRPGs are considered: the first model is a two-area power grid that combines three conventional power plants and two RESs in each area, while the second model is the IEEE 39-bus system. The tie-line is connected in series with a unified power flow controller (UPFC). The first method introduces an approach in the secondary control loop (SCL), where a fuzzy logic controller is cascaded with an Integral-Tilt-Derivative (I-TD) controller (Fuzzy I-TD). Additionally, the performance of the Fuzzy I-TD controller is compared with other approaches, such as Fuzzy Proportional-Integral-Derivative (Fuzzy-PID) and Fuzzy Integral-Proportional-Derivative (Fuzzy I-PD). The second strategy integrates the Fuzzy I-TD controller in the SCL along with controlled energy storage systems (ESSs), such as plug-in electric vehicles (PEVs). The parameters of the strategies are optimized using a recent metaheuristic algorithm known as the Sea Horse Optimizer (SHO) under different operating conditions. A comprehensive investigation is conducted to validate the effectiveness of the Fuzzy I-TD controller and the Fuzzy I-TD controller with PEVs in HRPGs. The Fuzzy I-TD controller significantly reduces frequency and tie-line deviations in the SCL by 82.7% and 97.01%, respectively, when compared to the Fuzzy I-PD and Fuzzy-PID controllers. Moreover, the Fuzzy I-TD with PEVs reduces frequency fluctuations by 40% compared to the Fuzzy I-TD alone in the SCL. The results demonstrate that the presented strategy is efficient and effective for HRPGs.

## Introduction

### Background

In recent times, electrical power systems have been confronted with critical challenges due to energy crises and environmental issues. These challenges arise from the reliance on conventional energy sources (CESs) that utilize fossil fuels, leading to carbon emissions and contributing significantly to global warming. As a result, there is a growing emphasis on integrating (RESs) into power systems as a substitute for CESs. This shift is motivated by the need to address the increasing demand and improve the technical performance of these systems. Energy planners are now focusing on strategies to enhance the efficiency and sustainability of power systems by promoting the greater adoption of RESs^1–3^. However, the inclusion of RESs in these systems reduces their inertia and may have a negative impact on the systems’ technical performance and stability due to the stochastic climatic variations of these sources, leading to numerous operational challenges related to the systems’ frequency stability^4–6^. Consequently, it is essential to maintain the system’s stability during the above-mentioned situations related to RESs by using load frequency control (LFC) on these systems in order to preserve the frequency at nominal value as well as the tie-line power of these power systems at their acceptable values^7–9^. Furthermore, the implementation of different energy storages in these systems, like PEVs as well as fuel cell systems (FCSs), which acts a necessary function in supporting the control capabilities and improving the system’s performance by injecting additional real power into the power grid^10–13^. Also, when two or more adjacent areas are connected, the flexible alternating current transmission system (FACTS) is widely applied with LFC to improve system stability. In this regard, more than one type of device has been applied to different systems to improve frequency, besides LFC^14,15^.

### State-of-the-art review

Numerous power system topologies, including single-area and multi-area interconnected systems as well as deregulated systems without and with consideration of nonlinearities have been addressed during the investigation of the frequency stability problem^16–18^. Several research studies have been investigated in order to implement different control methods including optimal control methods^10,19^, model predictive control (MPC)^20^, robust control methods^21–23^, and intelligent control techniques such as fuzzy logic control (FLC) and artificial neural network (ANN)^24–26^ for enhancing frequency stability and tie-line power transfer of the electrical systems within permissible ranges.

On the other hand, the most popular control method in electrical systems is yet the PID controller when compared against other controllers because of its best features including simple design and low cost^26^. However, the sensitivity of the PID controller to the uncertain climatic variations of RESs along with variable load demand represents the major drawback of this controller, which suffers from a complex estimation for their parameters^27^. Furthermore, fractional-order controllers (FOCs) are becoming increasingly popular in power systems because of their larger degree of freedom and flexible structure, which resists the nonlinearities as well as, it provides extra specifications to adjust because we have more changes of damped poles. From the above, the stability region has rapidly grown, providing us with greater design freedom for controller’s design^28–30^. Moreover, among FOCs, there are various research studies related to the tilt-integral-derivative (TID) controller for solving the problems of LFC have been demonstrated. The resilience, enhanced disturbance rejection capability, and flexibility to modify the closed-loop system’s parameters represent the major benefits of the TID controller. Also, more than arrangement has been investigated to enhance the performance of controllers, then improving the performance of the considered systems. Cascaded controller structures (CCS), combination structure (CS), and feed forward-feedback structure (FFS) have been considered the most popular arrangement. Firstly, the CCS has more merits such as better outcomes might be attained when the CCS has more tuning knobs than the non-crude CCS. Therefore, one of the best controller strategies for enhancing a control system’s defensive performance in control applications, especially in the event of disturbances is the CCS. In this regard the CCS has been applied in^31^ for damping the frequency fluctuations of these systems over the non-CCS. Secondly, the CS has more advantages such as maximizing the benefits of both controllers operating together, than one controller alone. A different approach was considered when designing the controller for LFC experiments. Regarding this, a combination of two controllers like a linear quadratic Gaussian controller combined with MPC controller^20^ as well as fuzzy combined with an MPC^32^. Thirdly, the FFS gives robustness to the controller gains where there is a change in reference input of controller. The I-PD and ID-T controllers consider as an example which give better performance than the traditional PID and TID controllers^33,34^. This signifies the authors’ inspiration to use the FFS arrangement to design a novel control strategy to boost the frequency in hybrid renewable electrical power grid.

As regards the estimation of controller parameters, several research works related to traditional and developed optimization algorithms were addressed for optimizing the controller parameters that used in the problem of frequency stability. Previously, a few old-style optimization methods were applied to evaluate the best frequency controllers’ constraints in^35,36^. To estimate the best controller parameters, a fuzzy gain scheduling controller has been addressed in^37^. Nevertheless, these optimization techniques face different limitations including need for much iteration, failing in achieving minimum function, and dependence on the initial situations during the estimation process the best parameters. Consequently, the investigators investigated to overcome these difficulties by creating a developed optimization techniques including particle swarm algorithm^38^, genetic algorithm^39^, moth swarm algorithm^40^, lightning attachment and its improved algorithm^41^, firefly algorithm^42^, electro-search algorithm^43^, mountain gazelle optimizer^44^, Jaya algorithm^45^, brancle mating optimizer^46^, Modified Crow Search Algorithm^47^, a crow-search algorithm^48^, (A-DQN) optimizer^49^, a quassi Oppositional- Path Finder algorithm^50^, an advanced-Sine Cosine Algorithm^51^. Accordingly to the developed meta-heuristic techniques and the benefits of fuzzy logic control scheme, it signifies the authors inspiration to use a recent algorithm known as sea horse optimizer (SHO) to estimate the best control parameters of the suggested Fuzzy I-TD controller in hybrid renewable power grid.

### Research contributions

- A comparison of the motivation behind the present work with those of previously published studies is provided in Table 1.

**Table 1:** Comparison of the motivation of the current work to those of other published studies.

References	^5^	^9^	^13^	^14^	^33^	^34^	^43^	Present work
Type of Controller	PID/FOPID	2DOF- TID	3DOF (1 + PID)- FOPI	PID	I-PD	ITDF	I	Fuzzy I-TD
Controller Design Technique	An Eagle Strategy Arithmetic Optimization Algorithm	Improved Gradient Based Optimizer	Hybrid Equilibrium Optimizer-slime mould optimization	Trial and Error (in steps form)	Fitness Dependent Optimizer	Imperialist competitive algorithm	Electro-Search optimization	Sea Horse Optimizer (SHO)
High Penetration Levels of RESs	√	√	√	×	×	×	×	√
Additional Improvements Incorporation	×	×	×	×	×	×	×	Considered CPEVs, FCS
Real-time Validation Considering Various Load Patterns	×	×	×	×	×	×	×	IEEE-39 Bus System/SLD
Real-time Validation Considering High RESs Penetration	×	×	×	×	×	×	×	√
The effect of Facts devices	×	×	×	Thyristor controlled phase shifter and Ultra capacitor	×	×	×	Unified Power Flow Controller

√: It means that considered, ×: It means not-considered.

The main contributions of the present article are outlined as follows:

- Presenting a coordination strategy that utilizes robust LFC and controlled hybrid ESSs to enhance the performance of HRPGs.
- An effective controller called Fuzzy I-TD is presented for the secondary control loop (SCL) system. It effectively mitigates frequency deviations in different areas of the HRPGs.
- The developed strategy is fine-tuned using a recent optimization technique called SHO, which identifies the optimal frequency control variables.
- The design of the developed controllers considers the high penetration of RESs, such as wind and solar power, in two identical areas of the HRPGs. The capacity of RESs exceeds 50% of the examined power grid capacity.
- System performance is compared between the presented controller and other controllers, such as Fuzzy-PID and Fuzzy I-PD, in the SCL.
- Various scenarios are considered to demonstrate the superiority of the hybrid control ESSs during high integration of RESs in the grid.
- The performance of the system is assessed by considering the influence of the UPFC connected in series with the AC tie line during abnormal operation between adjacent areas in the HRPGs.
- The studied controller’s reliability is verified by applying it to the IEEE system with 39 buses.

### Paper organization

The following sections are arranged as: In Sect. 2, the model of the hybrid-RPGs under research is explained. Sect. 3 presents the Fuzzy I-TD controller configuration and the role of the SHO technique in choosing the parameters of the presented controller. Section 4 displays the simulation outcomes and their discussion. Finally, the conclusions are displayed in Sect. 5.

## Modeling of HRPGs

### HRPGs configuration under research study

Two HRPGs are considered in this present study, each of them combines three CESs including thermal, hydro, and gas turbines as well as two RESs including PV and wind power plants as illustrated in Fig. 1. Formerly; the simplified block diagram of the HRPGs is depicted in Fig. 2. Additionally, the all-considered relations for the blocks in Fig. 2 are listed in Table A.1 and A.2 of Appendix A, respectively. Furthermore, Table 2 lists the capacity of each generating unit in the considered HRPGs.

**Table 2:** The generating units’ capacity.

Generating unit	Capacity (MW)
Thermal unit	1087
Hydropower unit	653
Gas turbine	262
Wind turbine	850
PV power plant	500

The Area Control Error (*ACE*) is the controller’s input signal, and the specific supplementary control action for every power plant refers to the output signal. The goal of this process is to generate more active power to enhance the performance of the HRPG performance. Besides, the formulas below indicate the formulas used to calculate the *ACEs* for interlinked HRPGs^8,52^.

1 $$\:{ACE}_{1}\:=\:{\varDelta\:\text{P}}_{\text{t}\text{i}\text{e}\:1-2}\:+\:{B}_{1}{\varDelta\:\text{f}}_{1}$$

2 $$\:{ACE}_{1}\:=\:{\varDelta\:\text{P}}_{\text{t}\text{i}\text{e}\:1-2}\:+\:{B}_{2}{\varDelta\:\text{f}}_{2}$$

**Fig. 1:**
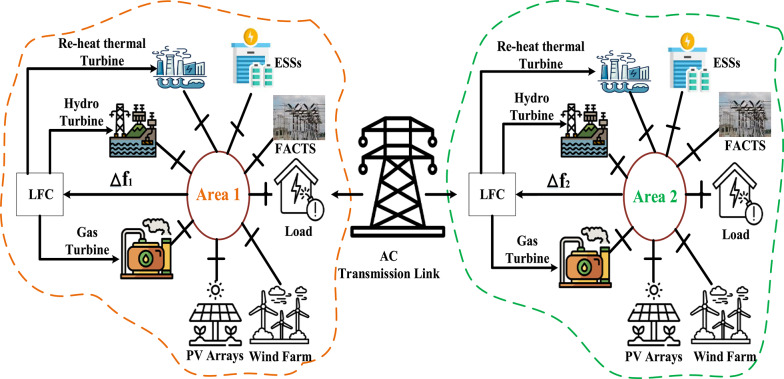
Schematic diagram of the HRPGs^12^.

**Fig. 2:**
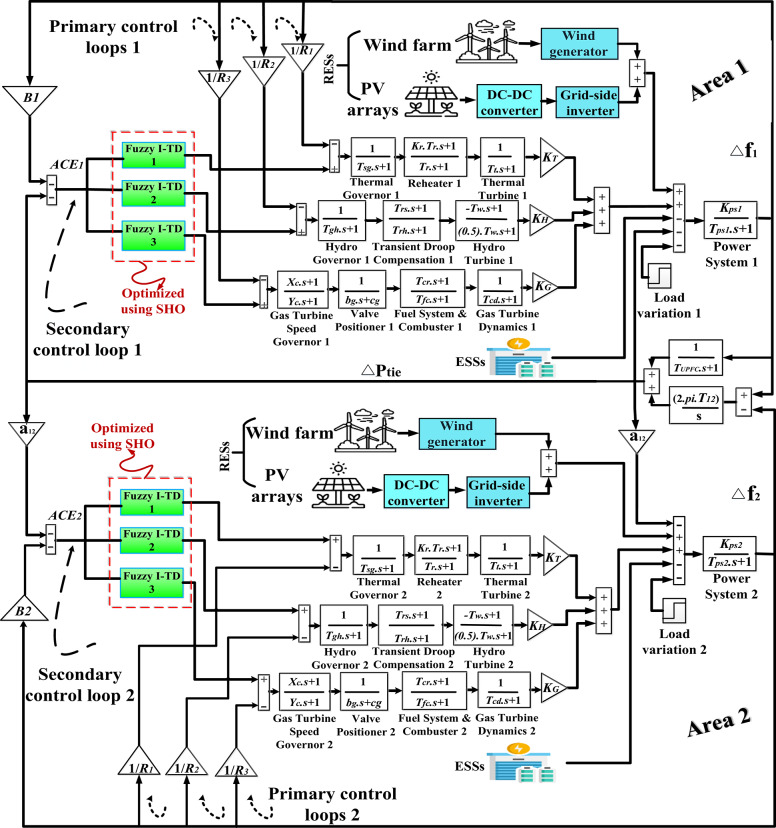
The simplified model of HRPGs^12,53^.

#### Conceptualization of the wind farm

**Fig. 3:**
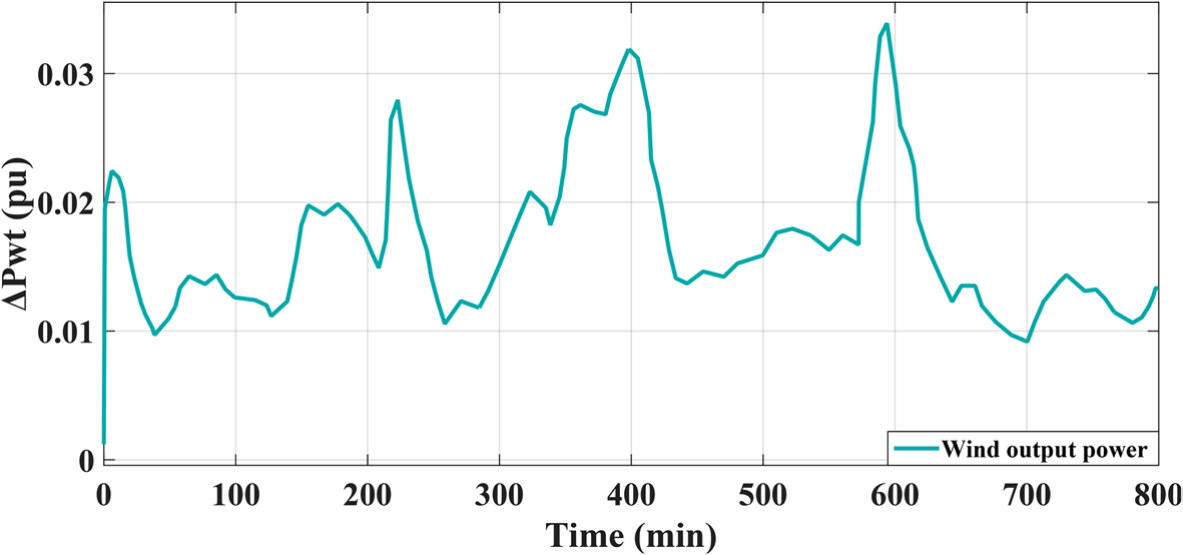
The output power of the wind station^8,54^.

The power of the installed wind plant $\:{P}_{wt}$ can be calculated by the following formula^8,54^,

3 $$\:{P}_{wt}=\:\frac{1}{2}\rho\:{A}_{T}{v}_{w}^{3}{C}_{p}\left(\lambda\:,\beta\:\right)$$

where the term $\:{C}_{p}\left({\lambda\:}_{i},\beta\:\right)$ can be represented as follows:

4 $$\:{C}_{p}\left({\lambda\:}_{i},\beta\:\right)=0.5\left({\lambda\:}_{i}-0.022{\beta\:}^{2}-5.6\right){\times\:e}^{-0.17{\lambda\:}_{i}}$$

5 $$\:{\lambda\:}_{i}=\frac{3600\times\:R\:}{1609\times\:\lambda\:}$$

6 $$\:\lambda\:=\frac{{\omega\:}_{B}\times\:R}{{V}_{W}}$$

where all parameters related to the installed wind units are described in Table A.3 of Appendix A. The transfer function, which represents the behavior of the integrated electric generator in a wind power plant, can be defined analytically to be a unity gain as well as a time constant of 0.3 s. Furthermore, the power output from wind plant is presented in Fig. 3.

#### Conceptualization of the PV station

The output power from the PV station can be expressed as follows^8,55^:

7 $$\:P={\eta\:}_{sc}{\tau\:}_{g}{\alpha\:}_{sc}RA\:[1-{\mu\:}_{sc}({T}_{sc}-{T}_{r}\left)\right]$$

**Fig. 4:**
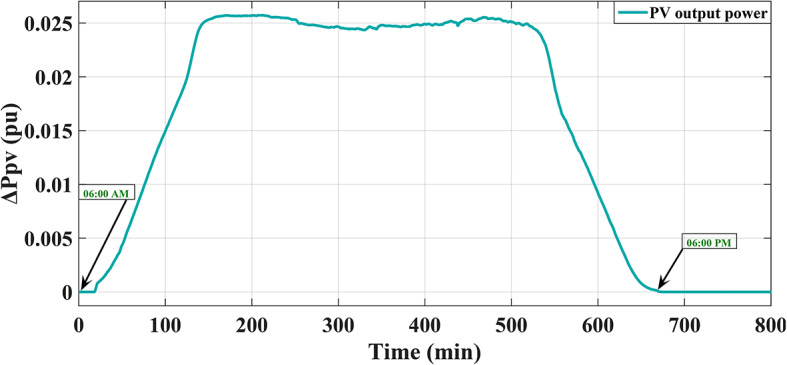
The output power of PV power plant^8^.

**Fig. 5:**
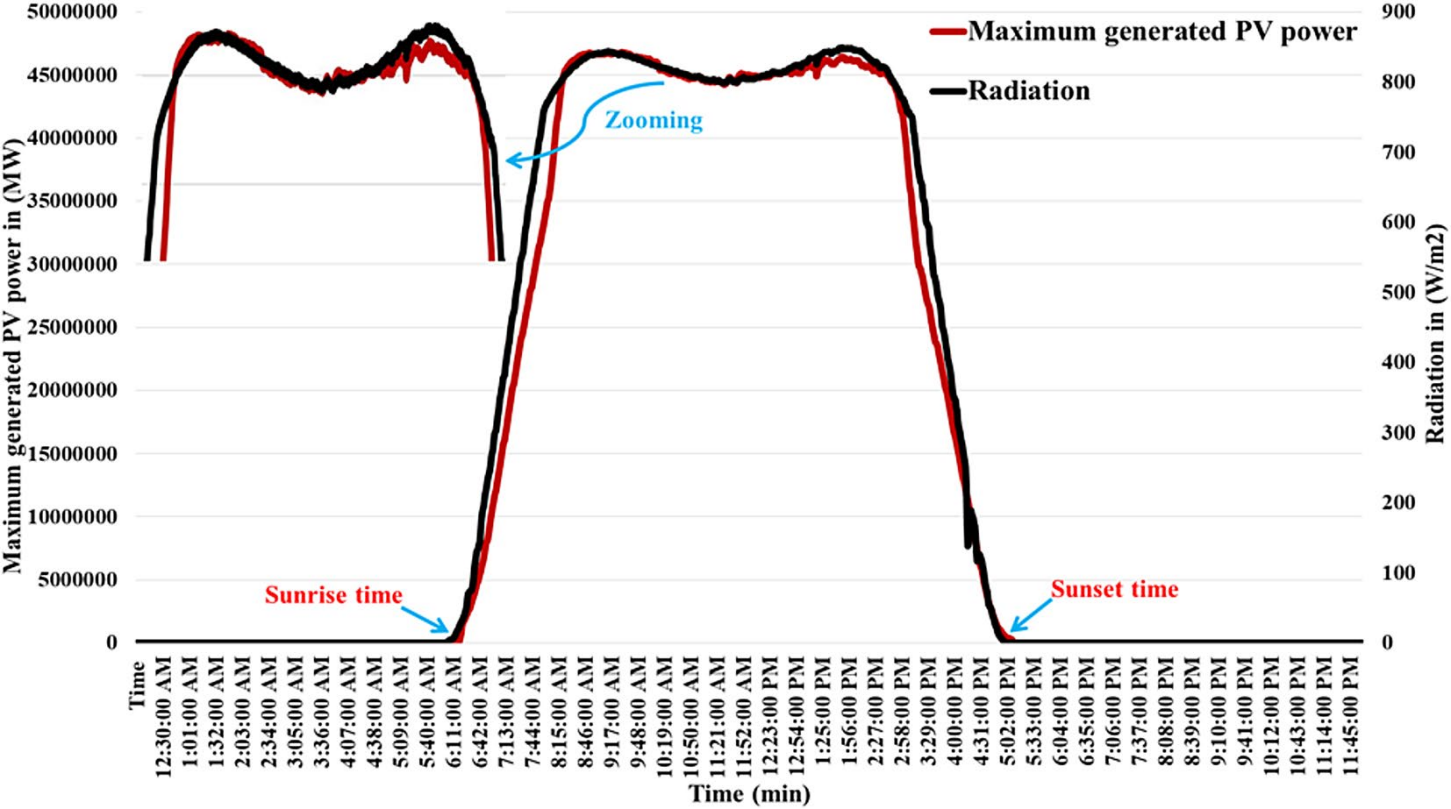
Features of the PV energy station’s power generation and radiation^8^.

**Fig. 6:**
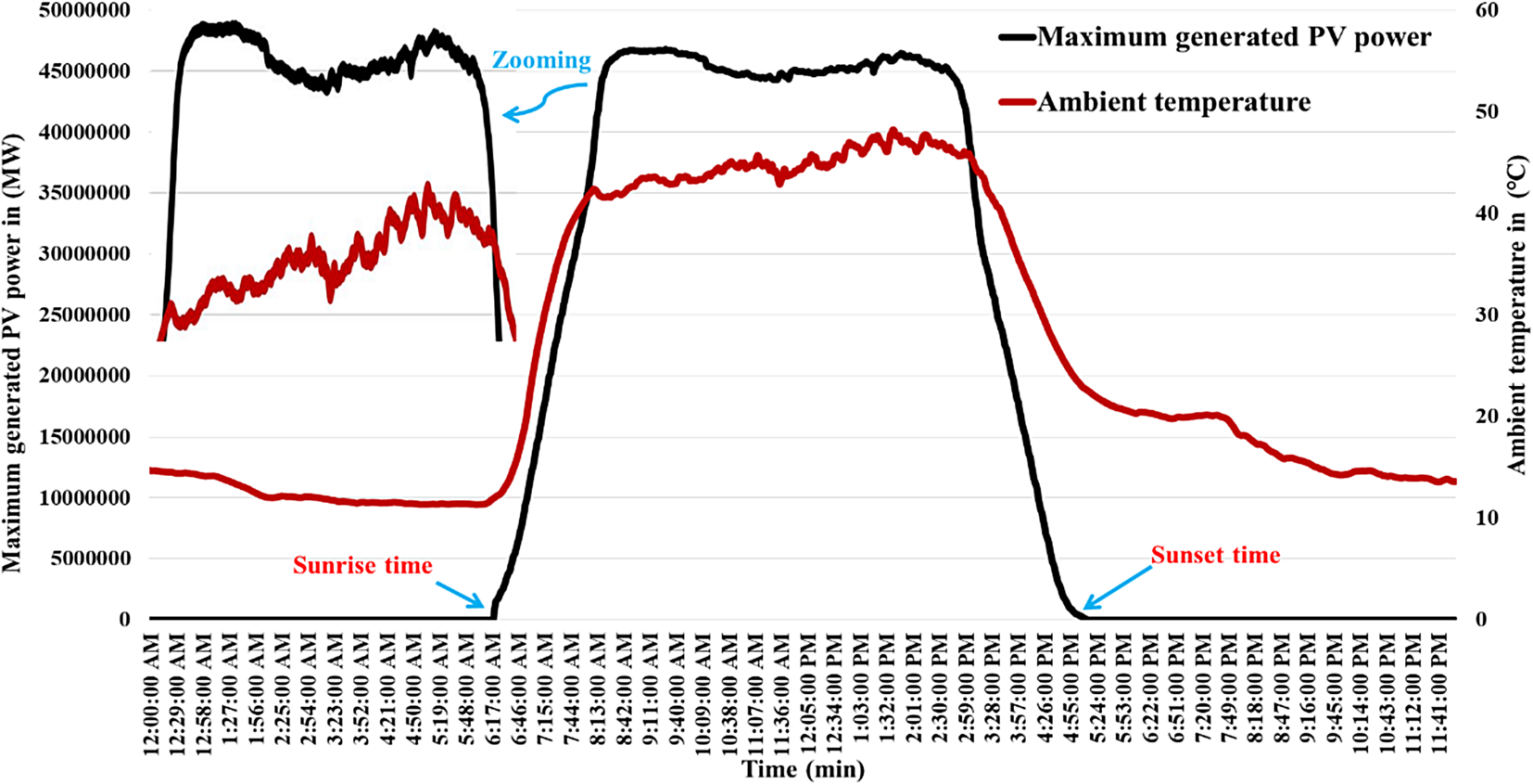
The PV energy station’s power generation characteristics and surrounding temperature^8^.

It is evident that varieties of factors, including the amount of solar radiation received, surface area of the PV cell, and the air temperature surrounding the surface, influence the generated power of a photovoltaic system. All parameters related to the PV plant are described in Table A.4 of Appendix A. The produced power of the PV plant is shown on Fig. 4. Table A.5 of Appendix A displays the temperature of the solar cells under various radiation conditions. In addition, the behavior of the maximum power of PV plant during the temporal variation of the radiation and ambient temperature is depicted in Figs. 5 and 6, respectively, for highlighting the relationships that exist between the manufactured active power, various levels of illumination, the manufactured active power, and the outside temperature of the surroundings.

#### Conceptualization of FCS in HRPGs

In this present study, the polymer electrolyte membrane fuel cell (PEMFC) is investigated to control the electrical grid’s frequency within allowable bounds due to its high-power density and fast response time^56^.

**Fig. 7:**
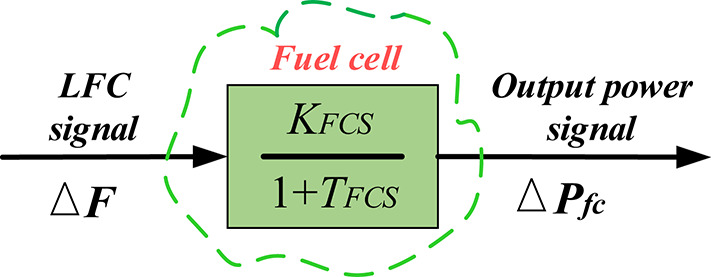
The investigated fuel cell model ^12,57^.

The electrolyzer and FC efficiency can be expressed as follows :

8 $$\:{\eta\:}_{F}=\frac{{P}_{F}}{{\dot{V}}_{F}HHV}$$

9 $$\:{\eta\:}_{e}=\frac{{\dot{V}}_{e}\:HHV}{{P}_{e}}$$

10 $$\:{V}_{H}={[V}_{H}^{0}+{\dot{V}}_{e}-{\dot{V}}_{F}]$$

where, $\:{P}_{F}$ and $\:{P}_{e}$ represent the Changing in fuel cell power, and the Changing in power consuming by electrolyzer, respectively, $\:{\dot{V}}_{e}$ and $\:{\dot{V}}_{F}$ categorize Electrolyzer operation causes a net change in hydrogen volume and transition in hydrogen volume due to the procedure of fuel cells respectively. The total amount and initial amount of hydrogen volumes of the tank are represented by $\:{V}_{H}$ and $\:{V}_{H}^{0}$ respectively. Also, $\:{\eta\:}_{e}$ and $\:{\eta\:}_{F}$ represent the electrolyzer and FC efficiency, respectively. Finally, $\:HHV$ is Greater warming value of hydrogen. Furthermore, Fig. 7 shows the variation performance of system frequency difference and FC power as follows^56^:

11 $$\:{\varDelta\:P}_{FC}\:=\:\left(\frac{{K}_{FC}}{1+S{T}_{FC}}\right)\:\varDelta\:F$$

where, the FC gains $\:{K}_{FC}$ equivalent to 0.03 and the time constant of the FC $\:{T}_{FC}$ equivalent to 3 s.

#### Conceptualization of PEV in HRPGs

Many studies have recommended the use of PEVs to mitigate the mismatch between generation and load demand in the presence of renewable energy sources (RESs)^58^. Consequently, by using comparable PEVs with a different inverter capability, the computational behavior of the PEVs can be regulated.

PEVs play a significant role in balancing generation and demand in RESs-integrated systems through their ability to function as flexible energy storage devices. This is achieved primarily via Vehicle-to-Grid (V2G) technology, which allows PEVs to not only consume energy but also feed it back into the grid when needed.

- PEVs can charge during periods of excess renewable energy generation and discharge during peak demand periods. This helps to flatten the load curve and reduce the need for additional peak-generation capacity.
- PEVs can provide grid services such as frequency regulation and voltage support. By adjusting their charging and discharging patterns in real-time, PEVs can help stabilize the grid in response to fluctuations in renewable energy generation.
- The batteries in PEVs function as distributed energy storage systems, absorbing surplus energy when RES generation exceeds demand and releasing it when demand exceeds generation. This mitigates the intermittency and variability of RESs.
- By storing excess renewable energy that would otherwise be curtailed due to grid constraints, PEVs enhance the utilization of RESs and reduce energy waste.

**Fig. 8:**
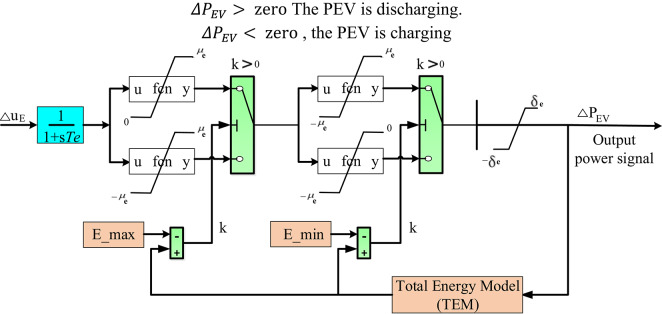
The investigated PEV model.

Furthermore, Fig. 8 displays the appropriate modeling of PEVs for the frequency stability study via its charging and discharging powers in a controllable situation^59,60^. According to Fig. 8, $\:{\varDelta\:u}_{E}$ represents the output signal from the HRPG area that can be used as the input to the PEV, $\:{T}_{e}$ characterizes the constant time of the PEV, $\:{\pm\:\mu\:}_{e}$ characterize the capacity constraint of the inverter, and $\:{\pm\:\delta\:}_{e}$ signifies the higher and lower limits of the power ramp rates. Additionally, $\:E$ characterizes the battery’s capacity, while the maximum and minimum rates of the controlled energy from the battery are $\:{E}_{max}$and $\:{E}_{min}$, respectively. The $\:{\varDelta\:P}_{EV}$ represents charging or discharging power^8^. Then, the $\:{\varDelta\:P}_{EV}$ can be expressed as follows:

12 $$\:{\varDelta\:P}_{EV}=\frac{{K}_{e}}{{T}_{e}s+1}{\varDelta\:u}_{E}$$

The following conditions define the charging and discharging of PEVs:

13 $$\begin{gathered} ~{\text{If}}~~\Delta P_{{EV}} = z{\text{ero}},{\text{ the PEV is idle}}{\text{.}} \hfill \\ \Delta P_{{EV}}> z{\text{ero, the PEV is discharging}}. \hfill \\ \Delta P_{{EV}} < z{\text{ero}},{\text{ the PEV is charging}} \hfill \\ \end{gathered}$$

#### Conceptualization of the UPFC in HRPGs

**Fig. 9:**
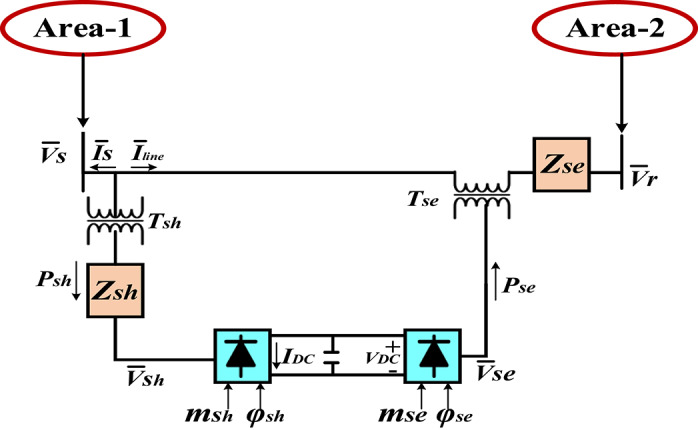
The connection between the two areas in HRPGs with UPFC.

**Fig. 10:**
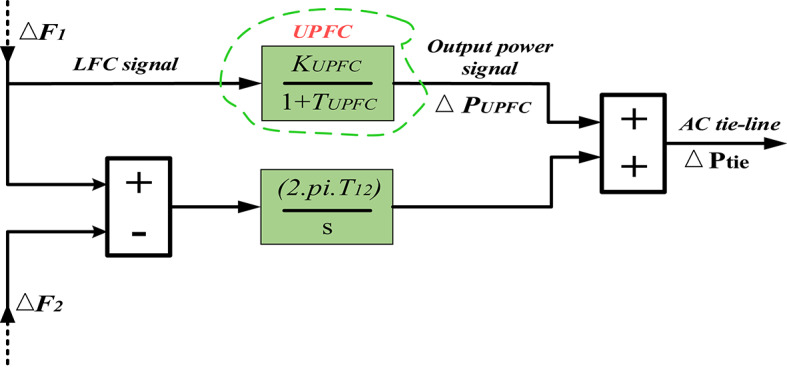
The investigated UPFC model in HRPGs^61^.

In the present study, the UPFC is used to adjust power flow, enhance transient stability, and maintain voltage stability. The UPFC is connected in series with a tie-line for damping the tie-line power oscillations. For instance, the load demand exchanges unexpectedly, the performance of LFC of generation logics are extremely disrupted and hence UPFC is established in series linking to tie-lines with the purpose of damp out the HRPGs fluctuations as rapidly as possible. The two areas interconnected power grids with UPFC is shown on Fig. 9. Furthermore, the investigation model of UPFC is shown on Fig. 10. The shunt converter supplied for the UPFC incorporates an adjustable shunt voltage, ensuring that the real or active portion of the current in the shunt branch balances the real power demanded by the series converter. The complex power on the receiving side of the line is stated as follows:

14 $$\:\:\:\:\:\:\:\:\:\:\:\:\:\:\:\:\:\:\:\:\:\:\:\:\:\:\:\:\:\:\:\:\:\:\:\:\:\:\:{P}_{\text{real}}-j{Q}_{\text{reactive}}={\stackrel{-}{V}}_{r}*{I}_{\text{line}}={\stackrel{-}{V}}_{r}*\left\{\left({\stackrel{-}{V}}_{\text{s}}+{\stackrel{-}{V}}_{\text{se}}-{\stackrel{-}{V}}_{r}\right)/j\left(X\right)\right\}$$

where,

15 $$\:{\stackrel{-}{V}}_{se}=\left|{V}_{se}\right|{\angle}\left({\delta\:}_{s}-{\phi\:}_{se}\right)$$

In previous equation, $\:{V}_{se}\:$refers to the magnitude of series voltage however $\:{\phi\:}_{se}$refers to the series voltage phase angle. The $\:{P}_{\text{real}}$ can be expressed as follows:

16 $$\:{\:P}_{\text{real}}=\frac{\left|{V}_{s}\right|\left|{V}_{r}\right|}{X}\text{sin}\delta\:+\frac{\left|{V}_{s}\right|\left|{V}_{se}\right|}{X}\text{sin}\left(\delta\:-{\phi\:}_{\text{se}}\right)={P}_{0}\left(\delta\:\right)+{P}_{\text{se}}\left(\delta\:,{\phi\:}_{\text{se}}\right)$$

If the $\:{V}_{se}$ value equal to zero, the above equation indicates an uncompensated real power system. In contrast, the UPFC series voltage magnitude may be regulated between 0 and$\:{\:\:V}_{se}\:max$, and its phase angle $\:{\phi\:}_{se}$may be adjusted from 0 to 360° at any power angle.

Furthermore, the power generated by the output signal of the UPFC could be described as below^61^. When the UPFC is connected in series with a tie-line to damp tie-line power oscillations. This is achieved through dynamic power compensation and modulation of series voltage injections, which alters the effective impedance of the tie-line and regulates power flow. The UPFC employs a damp control loop that monitors oscillations and generates appropriate modulation signals to counteract them, thereby enhancing system stability.

17 $$\:{\varDelta\:P}_{UPFC}=\left(\frac{{K}_{UPFC}}{1+S{T}_{UPFC}}\right){\Delta\:}\text{F}$$

## Problem identification and the controller structure

In this present study, the Fuzzy I-TD controller is investigated to minimize the fluctuations related to tie-line power and frequency of the system resulting from high penetration of RESs as well as the load disruptions while accounting for HRPDs nonlinearities. Furthermore, the optimal values of the Fuzzy I-TD controller are obtained using a novel optimization algorithm called SHO algorithm.

### The developed fuzzy I-TD controller

The controller combines the benefits of the fuzzy arrangement besides a modified design of the TID controller. Fuzzy I-TD leverages the benefits of fuzzy logic, fractional-order controllers, and feedback structures. As such, the fuzzy logic system can provide the most effective solution to complex issues and deal with systems uncertainties. Furthermore, a fractional-order controller is used due to its merits (i.e., more freedom, flexibility in structure, and having further adjustments of damping poles). As a result, the stability region has rapidly grown, providing us with greater design freedom for the controller’s design. Additionally, the feedback arrangement is utilized to rearrange the parameters of the TID controller to utilize the arrangement benefits (i.e., disturbance rejection, reduced lag, combined strengths, and versatility). According to the merits of fuzzy logic and fractional order schemes$\:,$ the proposed controller outperforms superior rejection of existing disturbances by dampening all low- and high-frequency disruptions. According to Fig. 11, the input for the construction of the proposed control technique is the *ACE*. The signal of the *ACE* is injected to the K_1_ and K_2,_ which represent the flexibility items for the inputs. Furthermore, the flexibility items for the result of the inputs can be symbolized by the gains of the improved TID. Detecting these scaling variables was previously difficult and frequently required methods of trial and error. This renders it difficult to identify the most effective parameters that boost system functionality. However, this research overcomes that limitation by proposing a Fuzzy I-TD controller. Moreover, these optimized values are identified using the SHO algorithm.

**Fig. 1:**
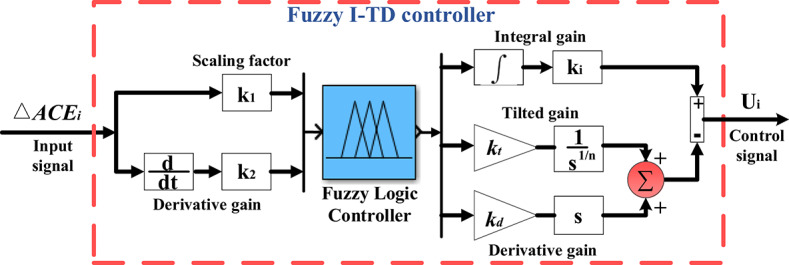
The Fuzzy I-TD controller structure.

The formula for mathematics associated with the TID controller is as outlined below^29^:

18 $$\:TID=\frac{{K}_{t}}{{S}^{\left(\frac{1}{n}\right)}}+\frac{{K}_{I}}{S}+{K}_{D}S$$

The gain values $\:{K}_{t},{K}_{i},$ and$\:{\:K}_{d}$ are selected among [0$\:,$ 10]$\:,$ $\:n$ is set between one and ten. Because of its simplicity and computational efficiency, triangular membership is frequently chosen in real-world electrical system applications. It is a reduction in computational load in first-order mathematics. Triangular membership functions show abundant symmetry in both input and output and are commonly used with I-TD controllers^62^.

Fuzzy control settings are chosen based on the unique properties of the system being considered as well as the designer’s level of experience. Predicting the input and output discursive universes of the system yields a range of fuzzy function memberships. The acceptable risk level for the input and output variables is usually chosen by the person who makes the decision. In this study, the range of time intervals is selected between − 1 and 1, as perturbations exceeding this range are not essential for achieving the stability of the system^63^.

**Fig. 12:**
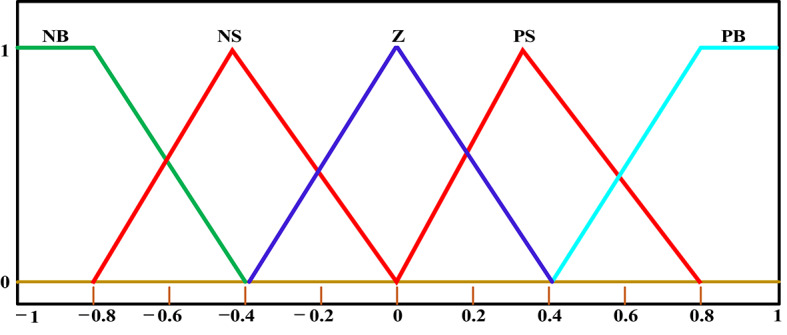
The triangular membership functions of the FLC’s input and output ^26,65^.

It is recommended to employ asymmetrical triangular functions for membership via 50% overlap; if necessary, a tuning procedure can be used to modify the overlap and spread to get the desired outcomes. Whereas, the triangular membership represents one of the attractive linear memberships of fuzzy methodology which characterized by less computation time and simplicity^64^. In the present study, the Fuzzy I-TD controller employs five triangle membership functions: negative big (NB), negative small (NS), zero (Z), positive small (PS), and positive big (PB)^26^. The membership functions are utilized for output as well as inputs, as depicted in Fig. 12. As a result, to produce fuzzy outputs, the Fuzzy-I-TD controller needs twenty-five rules, which are essential to its operation. Table 3 presents these rules and regulations.

**Table 3:** Rule base of the FLC.

$$\:\varvec{A}\varvec{C}\varvec{E}$$	$$\:\raisebox{1ex}{$\varvec{d}\varvec{A}\varvec{C}\varvec{E}$}\!\left/\:\!\raisebox{-1ex}{$\varvec{d}\varvec{t}$}\right.$$				
	NB	NS	Z	PS	PB
NB	NB	NB	NB	NS	Z
NS	NB	NB	NS	Z	PS
Z	NB	NS	Z	PS	PB
PS	NS	Z	PS	PB	PB
PB	Z	PS	PB	PB	PB

The integral time absolute error (ITAE) criterion is recommended to significantly reduce the value of the cost function. When a time-weighted term is added to the ITAE criterion, the optimization performance and system stability are enhanced compared to using the integral of squared error (ISE) criterion. When the time term is multiplied by the integral of the absolute error, error reduction becomes more effective. The following is the mathematical expression of the ITAE criterion^12^:

19 $$\:J=ITAE=\underset{0}{\overset{Tsim}{\int\:}}t.[\:\left|{\varDelta\:\text{f}}_{1\:}\right|\:+\:\left|{\varDelta\:\text{f}}_{2\:}\right|\:+\:\left|{\varDelta\:\text{p}}_{\text{t}\text{i}\text{e}1-2\:}\right|]\:dt$$

where *J* denotes the proposed cost function’s value, which aims to be as low as possible, $\:\left|{\varDelta\:\text{f}}_{1\:}\right|\:$signifies the area 1 frequency aberrations $\:,$ $\:\left|{\varDelta\:\text{f}}_{2\:}\right|$ signifies the area 2 frequency aberrations $\:,$ $\:\left|{\varDelta\:\text{p}}_{\text{t}\text{i}\text{e}1-2\:}\right|$ denotes the tie-line’s power flow change absolute error$\:,$ $\:Tsim$ signifies the overall simulation runtime$\:,$ and the variable $\:dt$ signifies the error signal sampling period through the simulation process.

Furthermore, the mathematical expression of Fuzzy I-TD controller is expressed according to the next equation:

20 $$\:\text{F}\text{u}\text{z}\text{z}\text{y}\:\text{I}-\text{T}\text{D}\:\left(s\right)=\:{K}_{1,i}+{K}_{2,i}s+\frac{{K}_{i,i}}{s}-(\frac{{K}_{ti,i}}{{S}^{\frac{1}{n}}}+{K}_{d,i}s)$$

The constraints of gain values$\:,{K}_{1,i(\text{F}\text{u}\text{z}\text{z}\text{y}\:\text{I}-\text{T}\text{D})},\:{K}_{2,i(\text{F}\text{u}\text{z}\text{z}\text{y}\:\text{I}-\text{T}\text{D})},\:{k}_{i,i(\text{F}\text{u}\text{z}\text{z}\text{y}\:\text{I}-\text{T}\text{D})},{k}_{ti,i(\text{F}\text{u}\text{z}\text{z}\text{y}\:\text{I}-\text{T}\text{D})}$, and $\:{k}_{d,i(\text{F}\text{u}\text{z}\text{z}\text{y}\:\text{I}-\text{T}\text{D})}$ utilized in this work in both areas of the considered power grid are expressed as follows:

21 $$\:{k}_{1,{i(\text{F}\text{u}\text{z}\text{z}\text{y}\:\text{I}-\text{T}\text{D})}^{max}}\ge\:{k}_{1,i(\text{F}\text{u}\text{z}\text{z}\text{y}\:\text{I}-\text{T}\text{D})}\ge\:{k}_{1,{i(\text{F}\text{u}\text{z}\text{z}\text{y}\:\text{I}-\text{T}\text{D})}^{min}}$$

22 $$\:{k}_{2,{i(\text{F}\text{u}\text{z}\text{z}\text{y}\:\text{I}-\text{T}\text{D})}^{max}}\ge\:{k}_{2,i(\text{F}\text{u}\text{z}\text{z}\text{y}\:\text{I}-\text{T}\text{D})}\ge\:{k}_{2,{i(\text{F}\text{u}\text{z}\text{z}\text{y}\:\text{I}-\text{T}\text{D})}^{min}}$$

23 $$\:{k}_{ti,{i(\text{F}\text{u}\text{z}\text{z}\text{y}\:\text{I}-\text{T}\text{D})}^{max}}\ge\:{k}_{ti,i(\text{F}\text{u}\text{z}\text{z}\text{y}\:\text{I}-\text{T}\text{D})}\ge\:{k}_{ti,{i(\text{F}\text{u}\text{z}\text{z}\text{y}\:\text{I}-\text{T}\text{D})}^{min}}$$

24 $$\:{k}_{i,{i(\text{F}\text{u}\text{z}\text{z}\text{y}\:\text{I}-\text{T}\text{D})}^{max}}\ge\:{k}_{i,i(\text{F}\text{u}\text{z}\text{z}\text{y}\:\text{I}-\text{T}\text{D})}\ge\:{k}_{i,{i(\text{F}\text{u}\text{z}\text{z}\text{y}\:\text{I}-\text{T}\text{D})}^{min}}$$

25 $$\:{k}_{d,{i(\text{F}\text{u}\text{z}\text{z}\text{y}\:\text{I}-\text{T}\text{D})}^{max}}\ge\:{k}_{d,i(\text{F}\text{u}\text{z}\text{z}\text{y}\:\text{I}-\text{T}\text{D})}\ge\:{k}_{d,{i(\text{F}\text{u}\text{z}\text{z}\text{y}\:\text{I}-\text{T}\text{D})}^{min}}$$

26 $$\:{n}_{{(\text{F}\text{u}\text{z}\text{z}\text{y}\:\text{I}-\text{T}\text{D})}^{max}}\ge\:{n}_{(\text{F}\text{u}\text{z}\text{z}\text{y}\:\text{I}-\text{T}\text{D})}\ge\:{n}_{{(\text{F}\text{u}\text{z}\text{z}\text{y}\:\text{I}-\text{T}\text{D})}^{min}}$$

where $\:i$ refers scheme of the suggested controller for each of the three mentioned traditional power plants; thus$\:,$ ($\:i$=1, 2, 3). The gain values $\:({k}_{1,i\left(\text{F}\text{u}\text{z}\text{z}\text{y}\:\text{I}-\text{T}\text{D}\right)},\:{k}_{2,i\left(\text{F}\text{u}\text{z}\text{z}\text{y}\:\text{I}-\text{T}\text{D}\right)},{k}_{ti,i\left(\text{F}\text{u}\text{z}\text{z}\text{y}\:\text{I}-\text{T}\text{D}\right)},{k}_{i,i\left(\text{F}\text{u}\text{z}\text{z}\text{y}\:\text{I}-\text{T}\text{D}\right)},$and $\:{k}_{d,i(\text{F}\text{u}\text{z}\text{z}\text{y}\:\text{I}-\text{T}\text{D})}$are chosen between [0$\:,$ 10]; $\:n$ is set among the range [1$\:,$ 10].

### The sea horse optimizer (SHO)

**Fig. 13:**
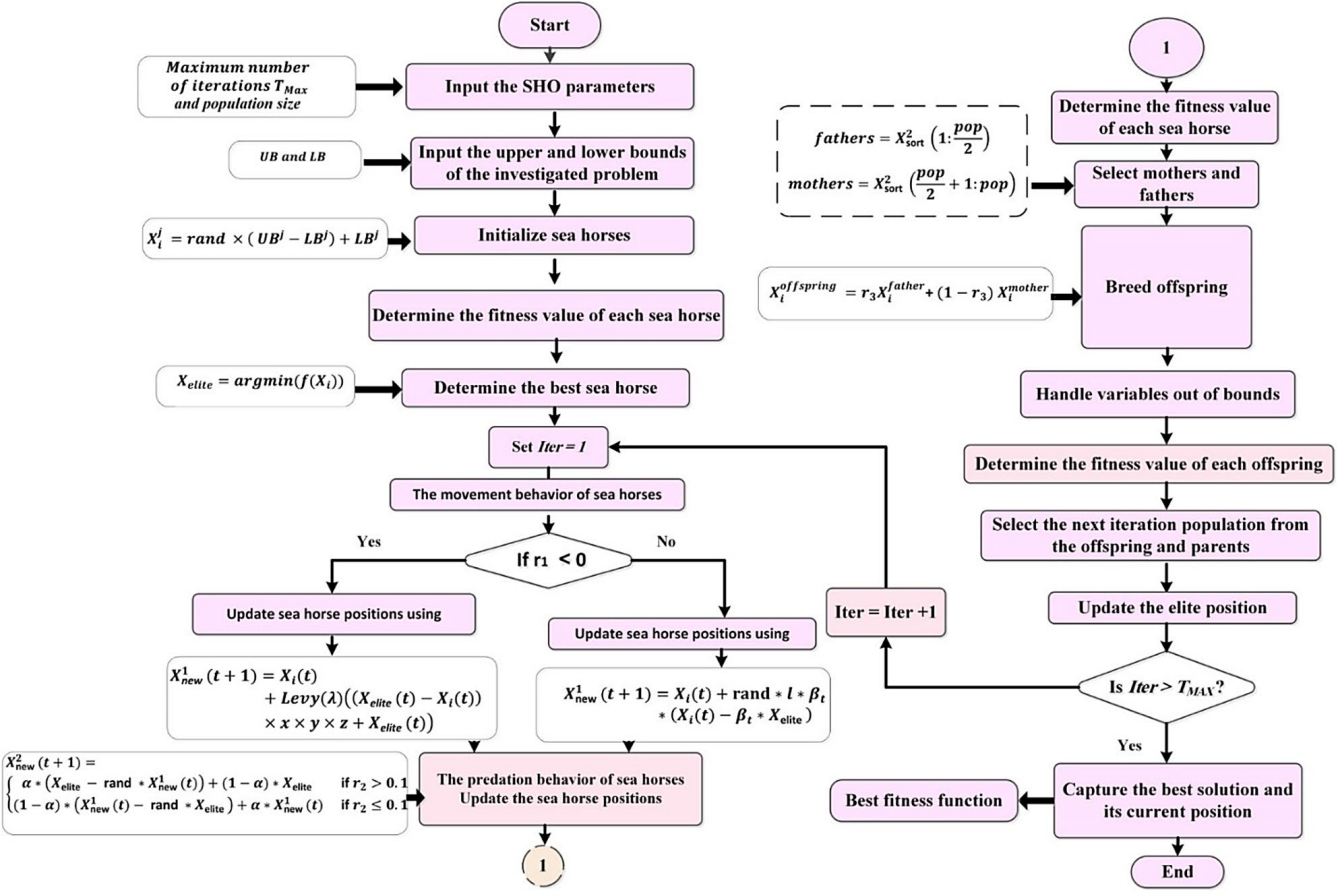
Flowchart of the applied SHO technique^66^.

In this present study, the SHO algorithm^66^ is used to select the optimal parameters of the Fuzzy I-TD. It is a recent algorithm existed based on natural heuristics, which simulates three diverse types of intelligent behaviors of sea horses, which are feeding, male reproduction, and movement. Figure 13 illustrates the flowchart of the SHO technique used for solving the studied optimization problem. 

## Simulation results and discussions

 In the present study, various scenarios and challenges are considered to assess and compare the performance of the proposed controller in hybrid renewable power grids (HRPGs). To ensure fair and accurate comparisons, all controllers are evaluated under identical conditions and operating environments.The optimization program for the SHO technique and the objective functions for the controllers are implemented using MATLAB’s m-file programming language. The SHO technique is executed with a maximum of 30 iterations, with a simulation time limit of 40 s, and 25 search agents are employed.

This section presents the validation and efficacy of the recommended Fuzzy I-TD controller, as well as the role of several ESSs, including FCS and PEVs, for boosting HRPGs frequency stability. Furthermore, the role of UPFC with PEVs as a type of ESSs to regulate the frequencies and power exchange among them in the examined HRPGs. The recommended Fuzzy I-TD controller’s durability is compared with effective and sophisticated control techniques, including Fuzzy-PID and Fuzzy I-PD under diverse operating conditions to validate its effectiveness and reliability, as discussed in the subsequent sections.

In this present study, various scenarios are investigated for addressing the HRPGs’ performance in terms of frequency and tie-line power deviations during different operating circumstances, as follows:

- Scenario No. 1: Assessing the Fuzzy I-TD controller in LFC at the two-area power grids considering SLP.
- Scenario No. 2: Assessing the Fuzzy I-TD controller in LFC at the two-area power grids considering SLD.
- Scenario No. 3: Assessing the Fuzzy I-TD controller in LFC at the two-area power grids considering random load disruption (RLD).
- Scenario No. 4: Assessing the Fuzzy I-TD controller in LFC at the two-area power grids considering renewables penetration.
- Scenario No. 5: Assessing the Fuzzy I-TD controller in LFC at the two-area power grids considering ESSs types along with FACTS.
- Scenario No. 6: Assessing the Fuzzy I-TD controller in LFC in the IEEE 39-bus system.
- Scenario No. 7: Assessing the strategy (Fuzzy I-TD with PEVs) in the modified IEEE 39-bus system.

### Scenario no. 1: assessing the fuzzy I-TD controller in LFC at the two-area power grids considering SLP

**Fig. 14:**
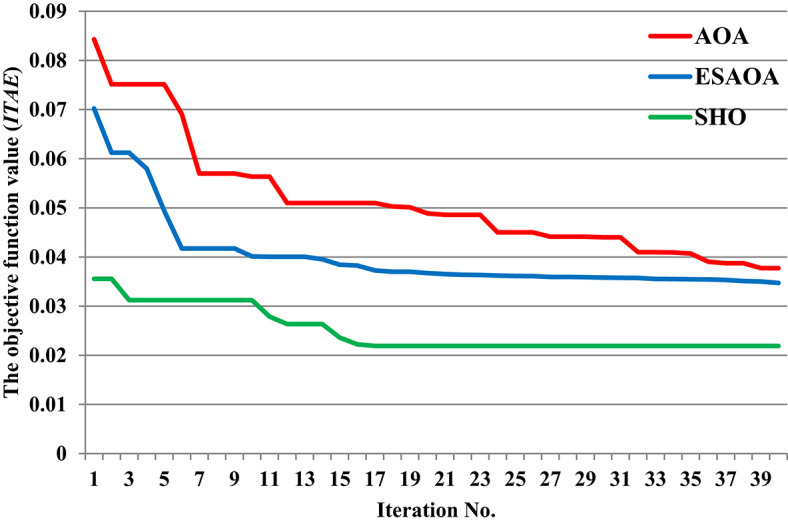
Convergence curve characteristics of the SHO optimizer and other considered optimizers.

This scenario is divided into two cases: the first focuses on validating the presented optimizer by comparing it with previous optimizers, while the second evaluates the proposed controller against traditional controllers. In the first case, a 1% SLD is applied to the first region in considered grid at t = 10 s. Figure 14 illustrates the convergence curve obtained, providing insights into the performance of the analyzed power grid when the recommended SHO optimizer. Additionally, Table 4 presents the optimal parameters obtained from the optimization process.

**Table 4:** Optimum coefficients of different optimizers considering PID controller in HRPGs.

Controller	Thermal plant	Hydro plant	Gas plant
PID based-TLBO	$\:{k}_{p}$= 4.1468, $\:{k}_{i}$= 4.0771, $\:{k}_{d}$= 2.0157	$\:{k}_{p}$= 1.0431, $\:{k}_{i}$= 0.6030, $\:{k}_{d}$= 2.2866	$\:{k}_{p}$= 4.7678, $\:{k}_{i}$= 3.7644, $\:{k}_{d}$= 4.9498
PID based-AOA	$\:{k}_{p}$= 10, $\:{k}_{i}$= 1.5975, $\:{k}_{d}$= 2.7449	$\:{k}_{p}$= 1.5975, $\:{k}_{i}$= 0.0837, $\:{k}_{d}$= 0.0875	$\:{k}_{p}$= 10, $\:{k}_{i}$= 10, $\:{k}_{d}$= 1.2779
PID based-ESAOA	$\:{k}_{p}$= 10, $\:{k}_{i}$= 1.4842, $\:{k}_{d}$= 6.1277	$\:{k}_{p}$= 9.6838, $\:{k}_{i}$= 0.0147, $\:{k}_{d}$= 0.3501	$\:{k}_{p}$= 1.4133, $\:{k}_{i}$= 9.8516, $\:{k}_{d}$= 0.1690
PID based-SHO	$\:{k}_{p}$= 10, $\:{k}_{i}$= 10, $\:{k}_{d}$= 5.445	$\:{k}_{p}$= 2.7374, $\:{k}_{i}$= 8.438, $\:{k}_{d}$= 2.511	$\:{k}_{p}$= 2.345, $\:{k}_{i}$= 1.098, $\:{k}_{d}$= 1.329

**Fig. 15:**
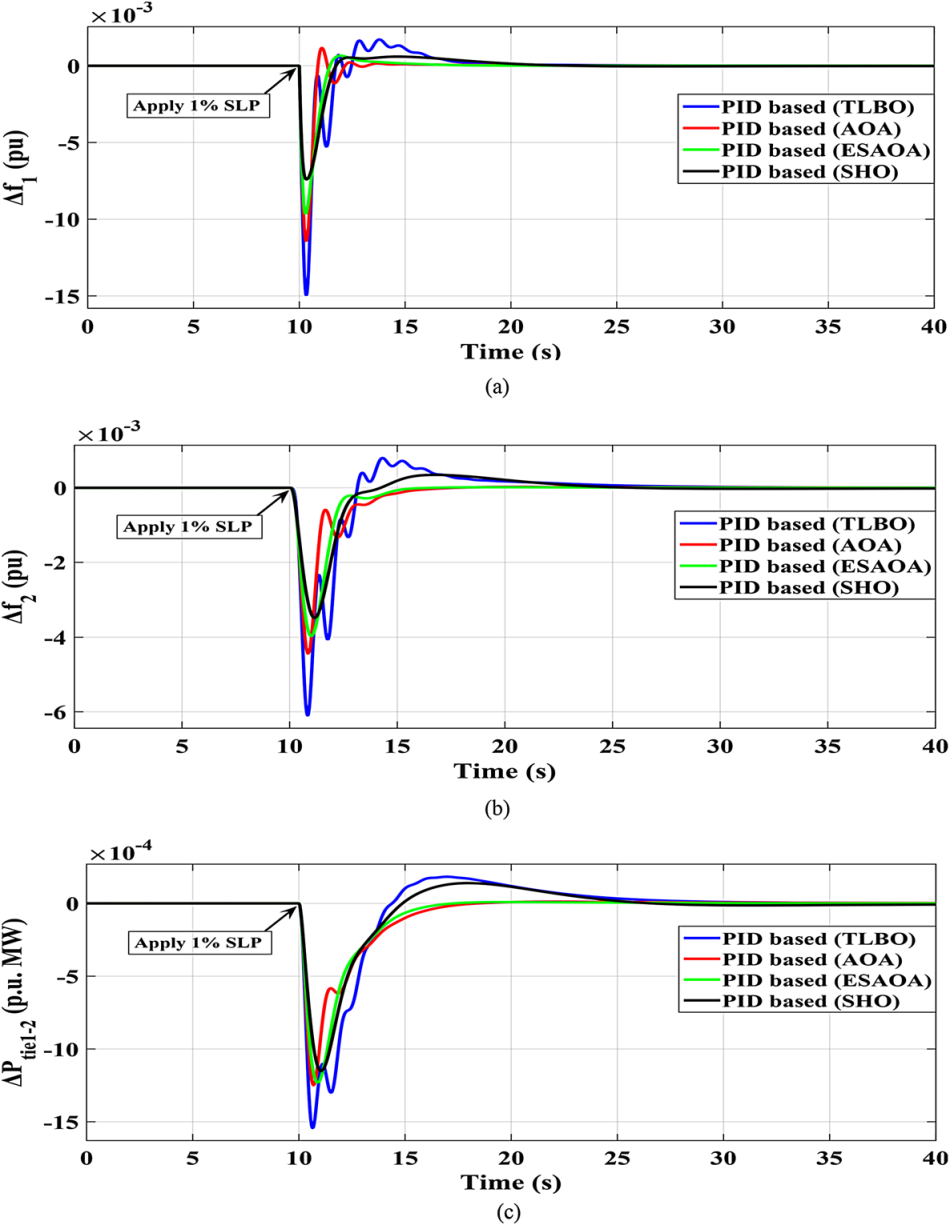
The HRPGs performance for case -1, Scenario-1 (a) $~\Delta {\text{f}}_{{1~}}$; (b) $~\Delta {\text{f}}_{{2~}}$; (c) $~\Delta {\text{p}}_{{{\text{tie}}1 - 2~}}$

**Fig. 16:**
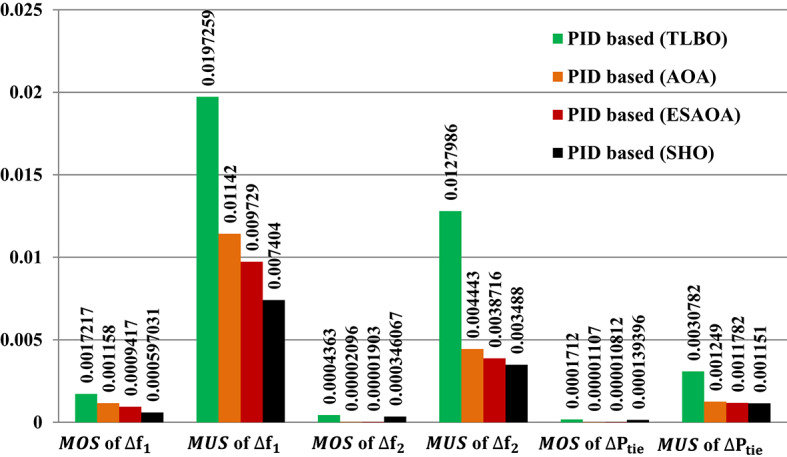
Performance evaluation obtained via the SHO optimizers besides various ones in Case 1, Scenario No. 1.

The response of HRPGs in terms of frequency and power deviations is shown on Fig. 15. The recommended SHO based PID controller system performance was compared to that of existing optimizers such as Teaching Learning-Based Optimization (TLBO), Arithmetic Optimization Algorithm (AOA), and an Eagle Strategy Arithmetic Optimization Algorithm (ESAOA)^9^. Furthermore, the obtained Performance evaluation for this scenario is depicted in Fig. 16.

**Fig. 17:**
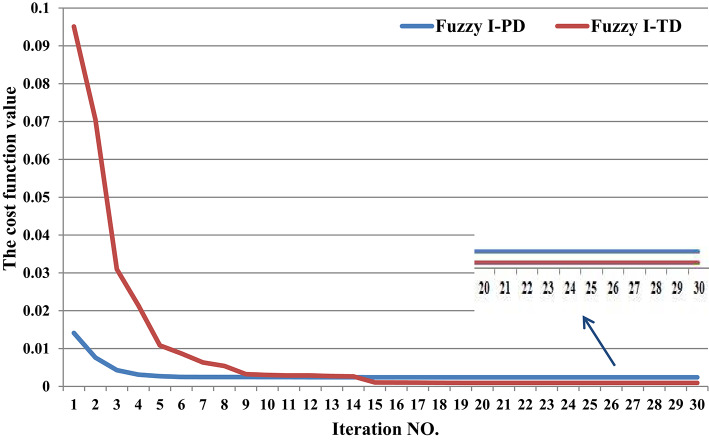
Convergence curve characteristics of the Fuzzy I-PD and Fuzzy I-TD optimized by the SHO technique.

In the second case, the robustness of the Fuzzy I-TD controller for LFC in HRPGs (i.e., two area power grid) without installed RESs. Furthermore, a 1% SLD is applied to first region in considered grid at t = 10 s, and a 1% SLD is applied to second region in considered grid at t = 20 s. Figure 17 illustrates the convergence curve obtained, providing insights into the performance of the analyzed power grid when the recommended controller, optimized using the SHO technique, is applied. Additionally, Table 5 presents the optimal parameters obtained from the optimization process.

**Table 5:** Optimum coefficients of different LFC controllers considering SLD in HRPGs.

Controller	Thermal plant	Hydro plant	Gas plant
Fuzzy-PID -AOA	$$\:{k}_{1}=\:10,\:{k}_{2}=\:4.7015,\:$$ $$\:{k}_{3}=\:4.7895,\:{k}_{4}=\:10$$	$$\:{k}_{1}$$= 10$$\:,$$$$\:{k}_{2}$$= 0.5402$$\:,$$ $$\:{k}_{3}=\:0.01,\:{k}_{4}=\:10$$	$$\:{k}_{1}=\:9.4636,\:{k}_{2}=\:10,\:$$ $$\:{k}_{3}=\:1.0988,\:{k}_{4}=\:10$$
Fuzzy I-PD - SHO	$$\:{k}_{1}$$= 10$$\:,$$$$\:{k}_{2}$$= 10$$\:,$$$$\:{k}_{p}$$= 9.9078, $$\:{k}_{i}$$= 10, $$\:{k}_{d}$$= 0.4354	$$\:{k}_{1}$$= 8.0151$$\:,$$$$\:{k}_{2}$$= 3.279$$\:,$$$$\:{k}_{p}$$= 0.349, $$\:{k}_{i}$$= 10, $$\:{k}_{d}$$= 3.1224	$$\:{k}_{1}$$= 4.46698$$\:,$$$$\:{k}_{2}$$= 4.1464$$\:,$$$$\:{k}_{p}$$= 10, $$\:{k}_{i}$$= 9.6768, $$\:{k}_{d}$$= 0.5783
Fuzzy I-TD - SHO	$$\:{k}_{1}$$= 9.1957$$\:,$$$$\:{k}_{2}$$= 0.4745$$\:,$$$$\:{k}_{t}$$= 4.0019, $$\:{k}_{i}$$= 7.7795, $$\:{k}_{d}$$= 3.3284, *n* = 5.4011	$$\:{k}_{1}$$= 0.0127$$\:,$$$$\:{k}_{2}$$= 0.8852$$\:,$$$$\:{k}_{t}$$= 8.0113, $$\:{k}_{i}$$= 0.27199, $$\:{k}_{d}$$= 1.1637, *n* = 9.0766	$$\:{k}_{1}$$= 9.1583$$\:,$$$$\:{k}_{2}$$= 1.9354$$\:,$$$$\:{k}_{t}$$= 6.3972, $$\:{k}_{i}$$= 0.4560, $$\:{k}_{d}$$= 0.51596, *n* = 7.27029

**Fig. 18:**
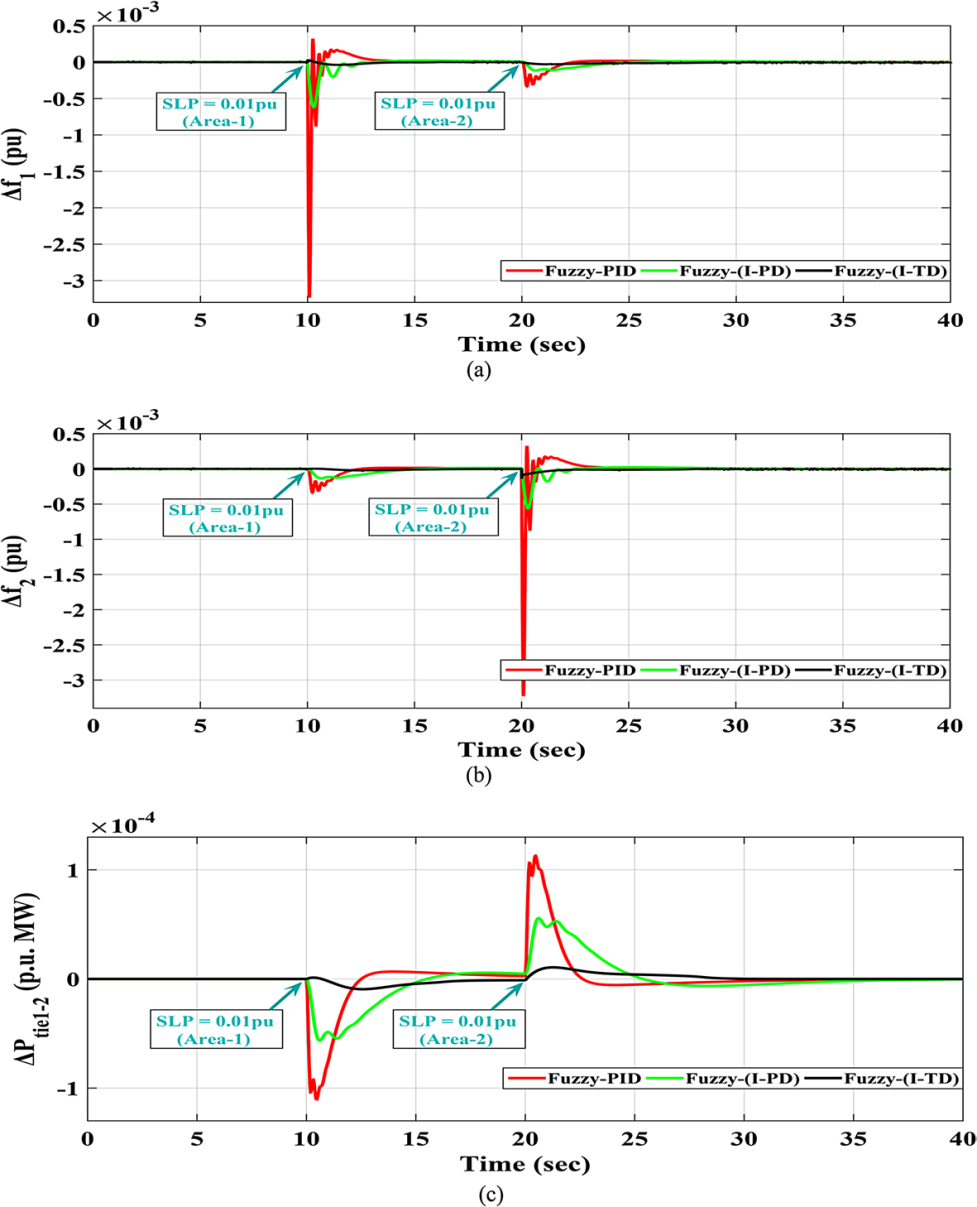
The Two areas PGs performance for Scenario-1 (a)$\:\:{\varDelta\:\text{f}}_{1\:}$; (b)$\:{\:\varDelta\:\text{f}}_{2\:}$; (c)$\:\:{\varDelta\:\text{p}}_{\text{t}\text{i}\text{e}1-2\:}$.

**Fig. 19:**
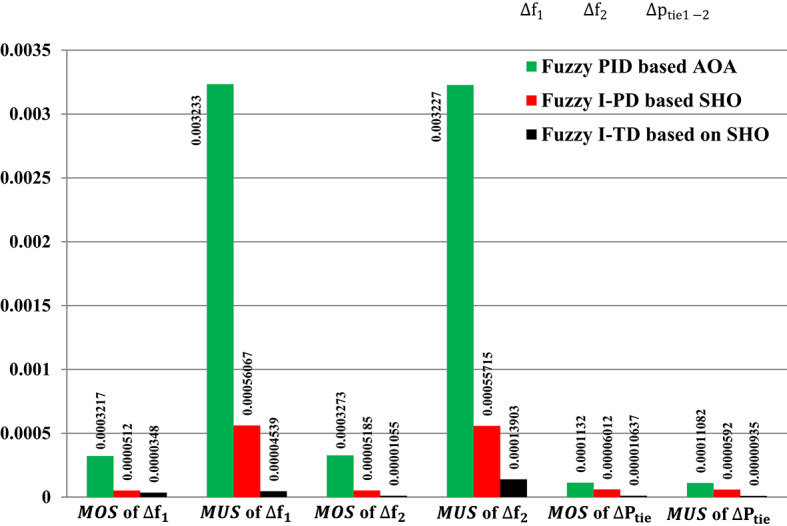
Performance evaluation obtained via the fuzzy I-TD controller besides various ones in Scenario No. 1.

**Fig. 20:**
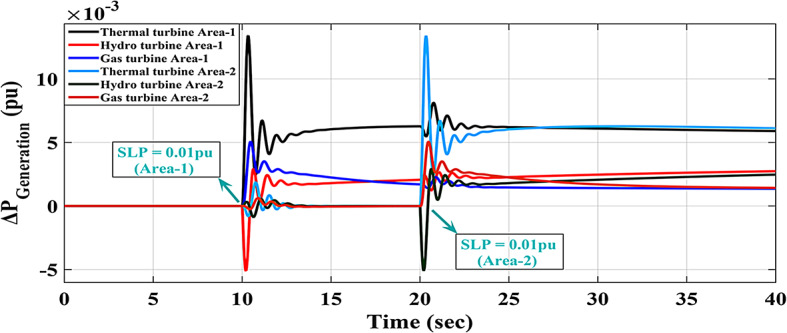
The Power generation curves for the two areas power grid for case-1, scenario 1.

The response of HRPGs in terms of frequency and power deviations is shown on Fig. 18. The recommended Fuzzy-I-TD controller’s system performance was compared to that of existing controllers such as Fuzzy-PID and Fuzzy I-PD. Furthermore, the obtained Performance evaluation for this scenario is depicted in Fig. 19. Additionally, the Power generation curves for this case are shown in Fig. 20.

The results depicted in Fig. 19 indicate that the Fuzzy-PID controller performs the least effectively, exhibiting high undershoot values in area 1, area 2, and tie-line power. Whereas these values are 0.003233 Hz in area 1, 0.003227 Hz in area 2, and 0.0001108 p.u. for tie-line power. However, the Fuzzy I-PD controller shows improvements by reducing the deviations to acceptable levels. However, the Fuzzy I-PD controller maintains frequency deviations of 0.00056067 Hz in area 1, 0.00055715 Hz in area 2, and 0.0000592 p.u. for power exchange. It maintains lower frequency deviations in both areas and reduces power exchange deviations compared to the earlier Fuzzy-PID controller results. On the other hand, the Fuzzy I-TD controller outperforms other controllers by swiftly and smoothly regulating frequency and power deviations with fewer oscillations. Whereas the recommended controller attains frequency deviations of 0.00004539 Hz in area 1, 0.00013903 Hz in area 2, and 0.00000935 p.u. for tie-line power. The recommended controller based on the Fuzzy I-TD approach achieves the smallest maximum overshoot ($\:MOS$) and maximum undershoot ($\:MUS$) for frequencies and exchange tie-line power in the dual areas, as evident from Fig. 19.

### Scenario no. 2: assessing the fuzzy I-TD controller in LFC at the two-area power grids considering SLD

**Fig. 21:**
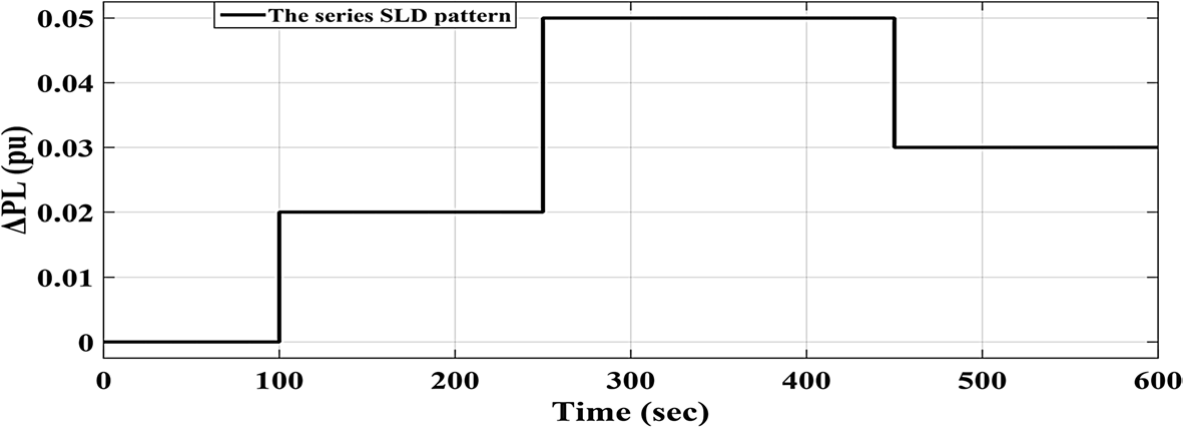
Applied series SLD profile.

**Fig. 22:**
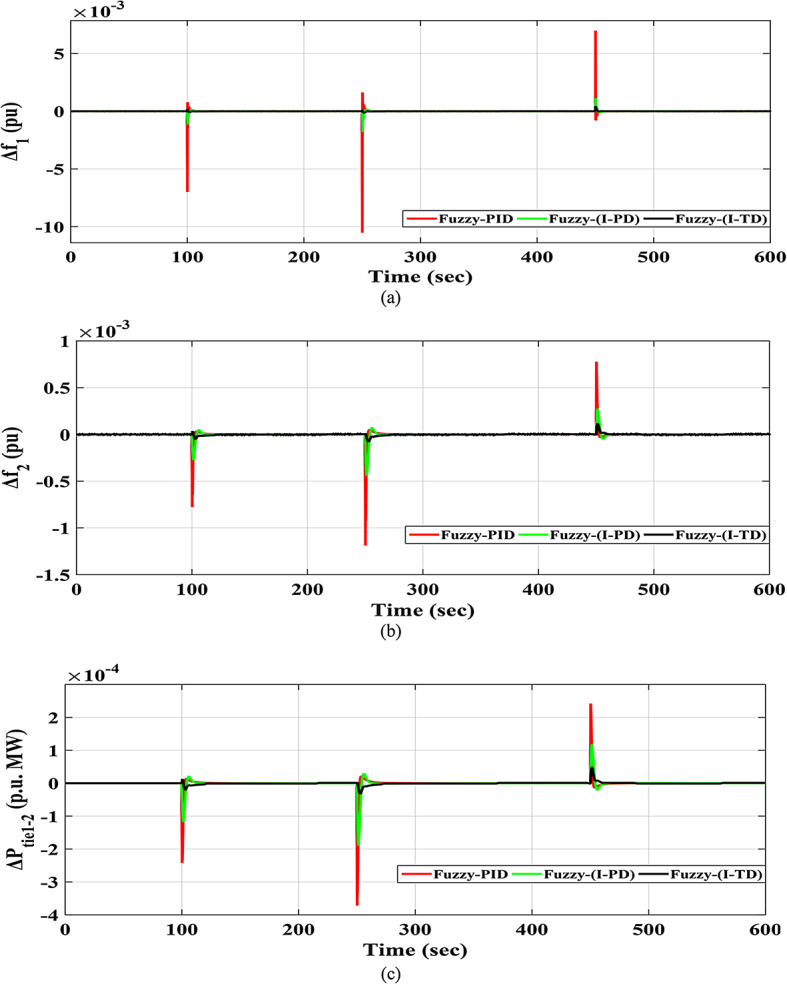
The HRPGs performance for Scenario-2 (a)$\:\:{\varDelta\:\text{f}}_{1\:}$; (b)$\:{\:\varDelta\:\text{f}}_{2\:}$; (c)$\:\:{\varDelta\:\text{p}}_{\text{t}\text{i}\text{e}1-2\:}$.

**Fig. 23:**
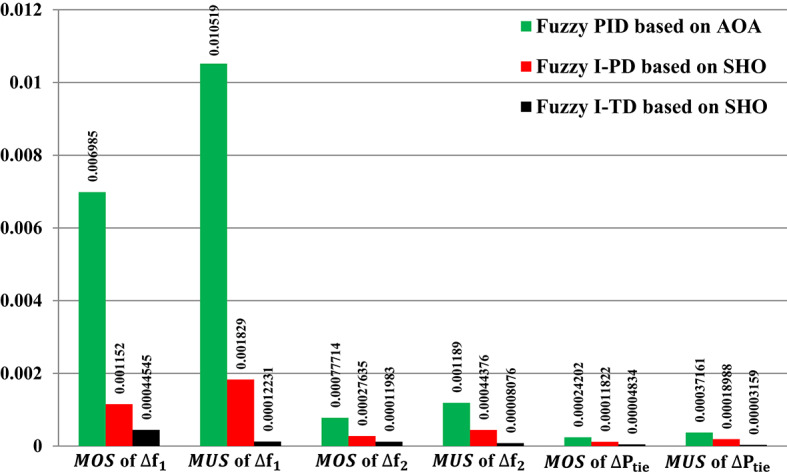
Performance evaluation obtained via the fuzzy I-TD controller besides various ones in Scenario No. 2.

**Fig. 24:**
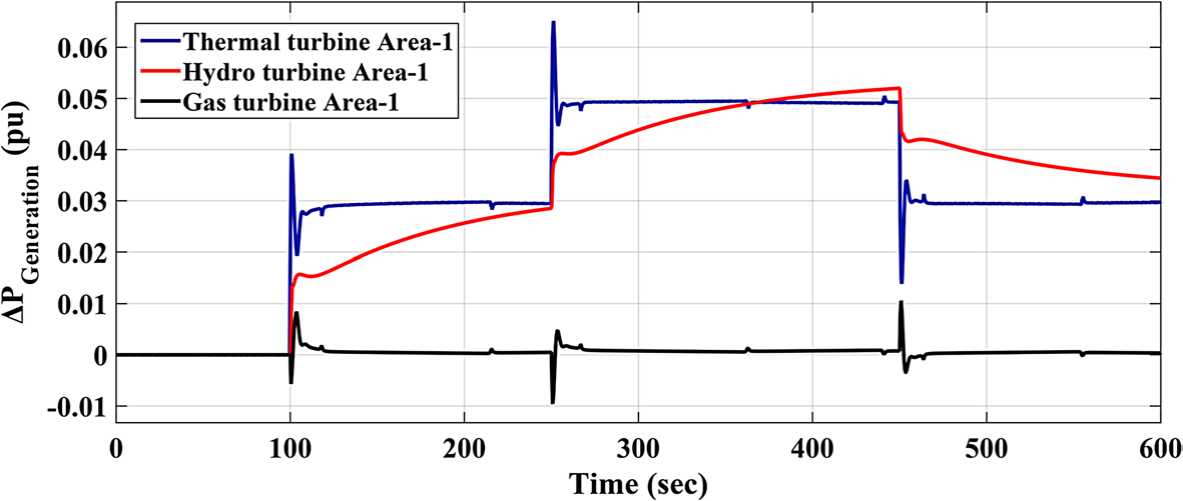
The Power generation curves for the two areas power grid for scenario 2.

The HRPGs experiences unpredictable series SLD in the first area of the investigated electric power grid as shown in Fig. 21. The main purpose is to evaluate the reliability of the recommended Fuzzy I-TD based on the SHO algorithm and compare it with Fuzzy-PID relied on AOA algorithm as well as Fuzzy I-PD relied on SHO algorithm. The HRPGs’ performance in the current scenario is depicted in Fig. 22. Furthermore, Fig. 23 demonstrates the outcomes of the performance evaluation for this scenario. Moreover, the Power generation curves for this case are shown in Fig. 24.

For instance, the Fuzzy-PID controller performs the least effectively with high overshoot values of 0.006985 Hz in area 1, 0.00077714 Hz in area 2, and 0.00024202 p.u. for tie-line power. The Fuzzy I-PD shows improvements compared to the Fuzzy-PID controller by suppressing fluctuations to acceptable frequencies. Whereas the Fuzzy I-PD controller maintains frequency deviations of 0.001152 Hz in area 1, 0.00027635 Hz in area 2, and 0.00011822 p.u. for power exchange. In addition, the proposed controller attains frequency deviations of 0.00044545 Hz in area 1, 0.00011983 Hz in area 2, and 0.00004834 p.u. for tie-line power. It is obvious that the recommended Fuzzy I-TD significantly diminishes the $\:MOS$ and $\:MUS$ of $\:{\varDelta\:\text{f}}_{1\:}$by 93.62% and 98.84% compared to the Fuzzy-PID controller.

### Scenario no. 3: assessing the fuzzy I-TD controller in LFC at the two-area power grids considering RLD

**Fig. 25:**
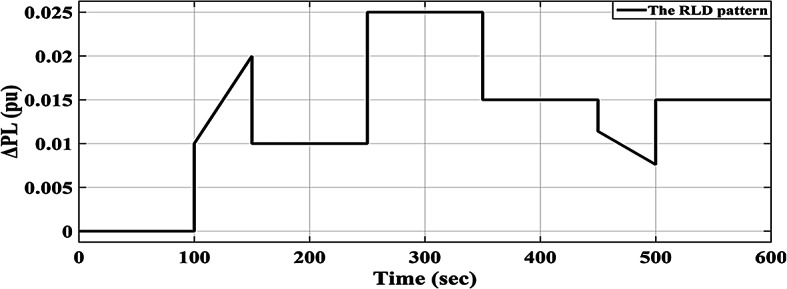
Applied RLD profile.

**Fig. 26:**
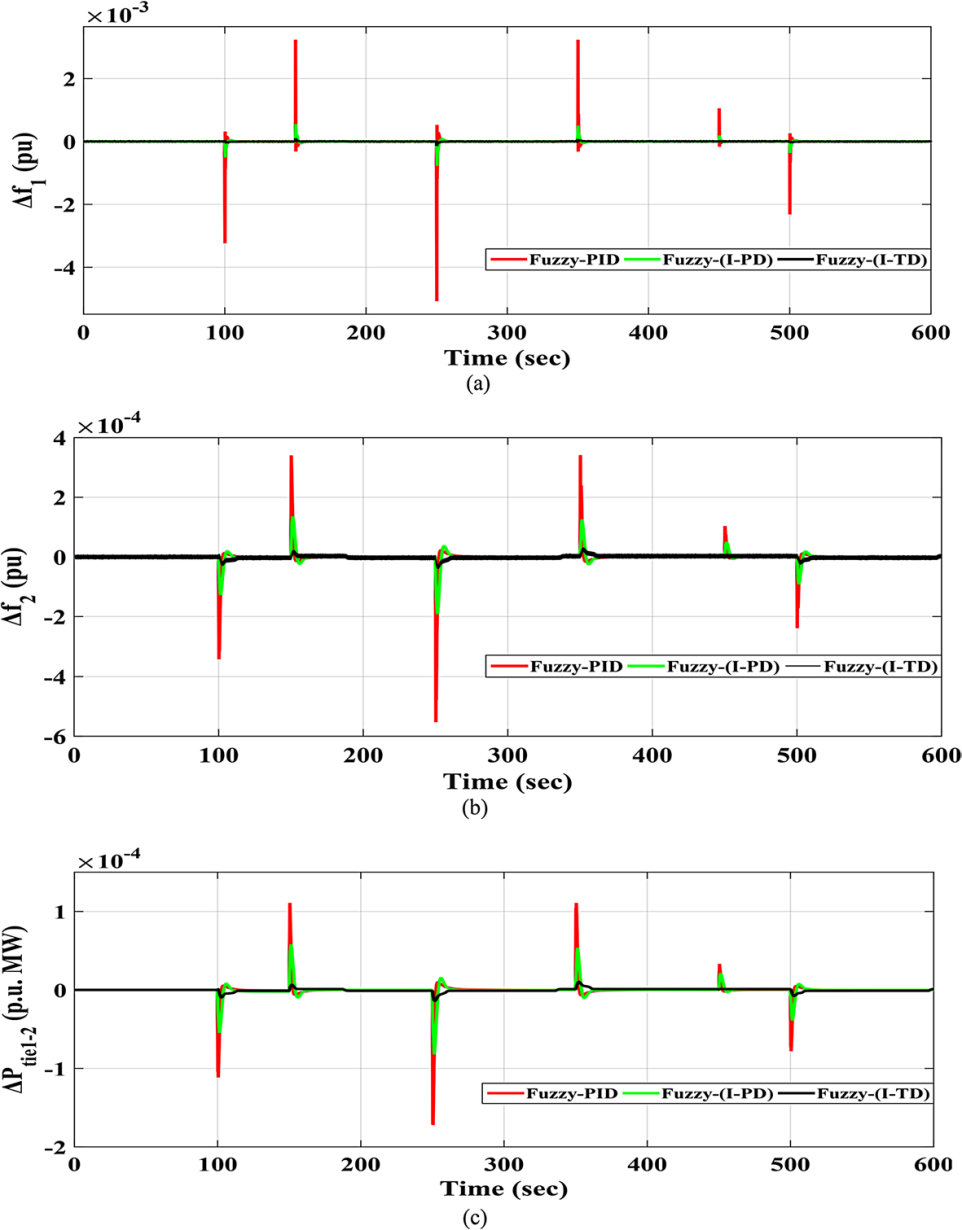
The HRPGs performance for Scenario-3 (a)$\:\:{\varDelta\:\text{f}}_{1\:}$; (b)$\:{\:\varDelta\:\text{f}}_{2\:}$; (c)$\:\:{\varDelta\:\text{p}}_{\text{t}\text{i}\text{e}1-2\:}$.

**Fig. 27:**
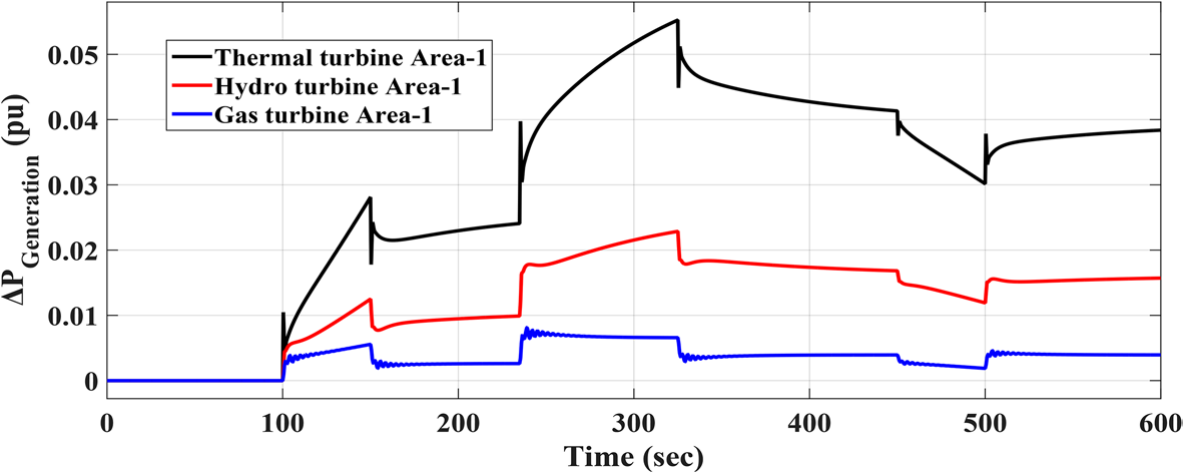
The Power generation curves for the two areas power grid for scenario 3.

A random load fluctuation occurring at t = 100 s is studied in the first area of the electric power grid investigated. This form represents a broad range of disruptions in industrially interconnected loads, resulting in similar grid effects such as grid instability and the occurrence of blackouts. The applicable RLD pattern is clarified in Fig. 25. Additionally, Fig. 26 presents a comparative analysis of the dynamic performance of the examined HRPDs. Moreover, the Power generation curves for this case are shown in Fig. 27.

**Fig. 28:**
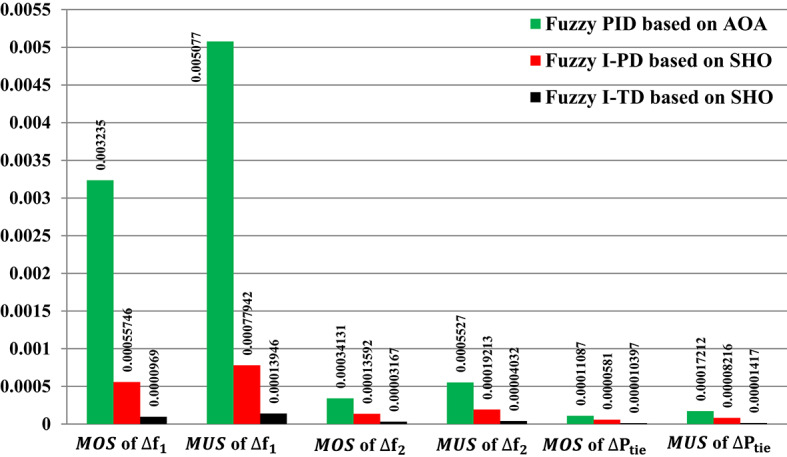
Performance evaluation obtained via the recommended controller besides various ones in Scenario No. 3.

Figure 28 shows the$\:\:MOS\:$and $\:MUS$ values for each of the both frequencies, as well as exchange tie-line power among both areas. Particularly, the Fuzzy-PID controller exhibits high undershoot values of 0.005077 Hz within area 1, 0.0005527 Hz within area 2, and 0.00017212 p.u. for tie-line power. The Fuzzy I-PD controller demonstrates improvements compared to the Fuzzy-PID controller by effectively dampening deviations to acceptable levels. Specifically, the Fuzzy I-PD controller maintains frequency deviations of 0.00077942 Hz within area 1, 0.00019213 Hz within area 2, and 0.00008216 p.u. for power exchange. Moreover, the considered controller achieves frequency deviations of 0.00013946 Hz within area 1, 0.00004032 Hz within area 2, and 0.00001417 p.u. for tie-line power. The recommended Fuzzy I-TD controller significantly reduces $\:MOS\:$and $\:MUS$ of $\:{\varDelta\:\text{f}}_{1\:}$by 97.00% and 97.25%, respectively, when compared to the Fuzzy-PID controller.

### Scenario no. 4: assessing the fuzzy I-TD controller in LFC at the two-area power grids considering renewables penetration

**Fig. 29:**
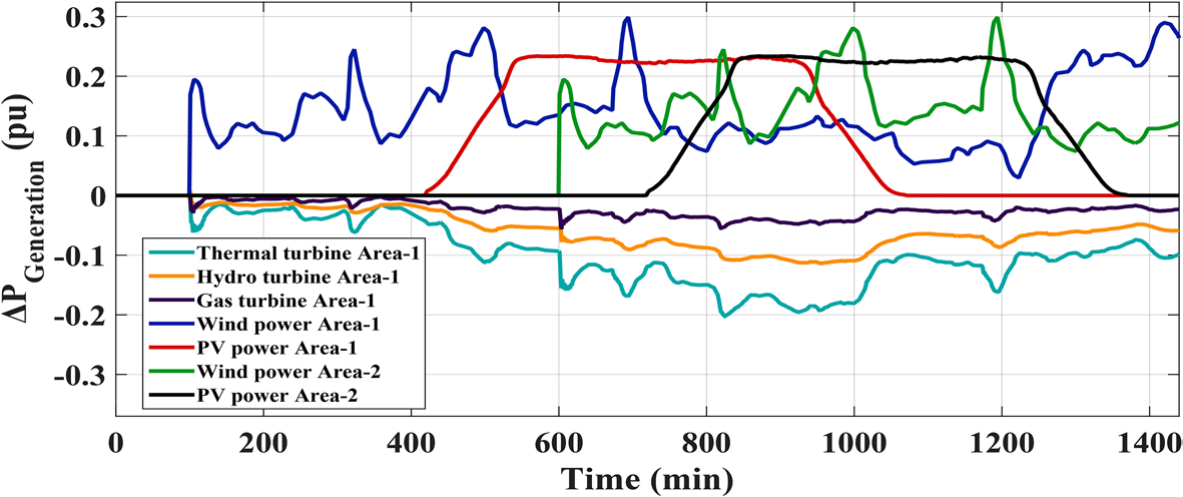
Output power of various RESs in HRPGs and the outputs of other generation sources for Scenario No. 4.

**Fig. 30:**
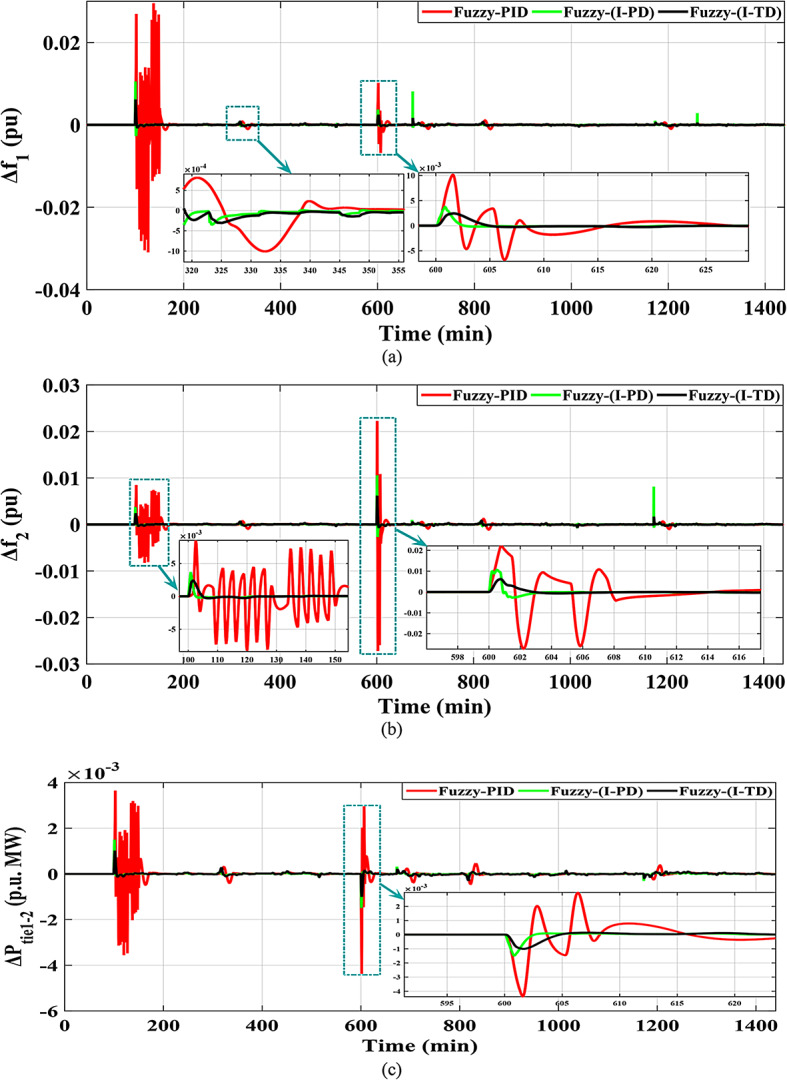
The HRPGs performance for Scenario-4 (a)$\:\:{\varDelta\:\text{f}}_{1\:}$; (b)$\:{\:\varDelta\:\text{f}}_{2\:}$; (c)$\:\:{\varDelta\:\text{p}}_{\text{t}\text{i}\text{e}1-2\:}$.

This scenario’s main objective is to investigate the disruptions induced by a significant penetration of renewables in both analyzed HRPGs. Specifically, ten wind farms and ten PV power plants were implemented to incorporate area 1 at 100 and 400 min, during the simulation time respectively, adopting a RLD pattern. While the renewables integration in area 2 adopts the same HRP integration environments; PV power plants integrate at 700 min and wind farms at 600 min. Furthermore, Figs. 29 and 30 demonstrate the power produced from the RESs integrated into the examined HRPGs and the other generation sources during renewables penetration, as well as various system dynamic responses.

**Fig. 31:**
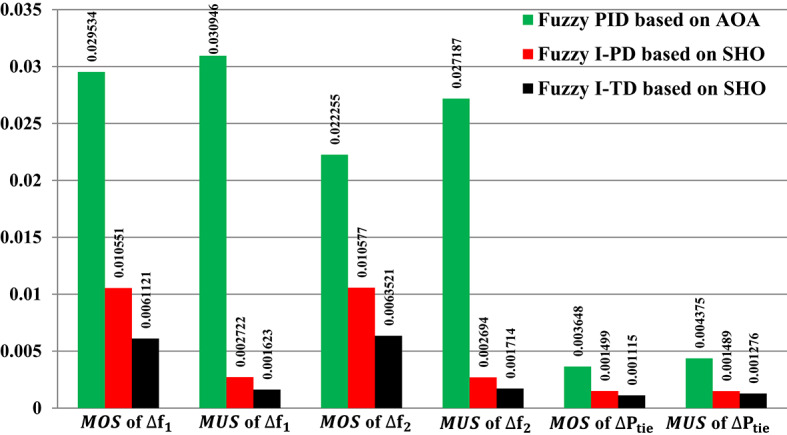
Performance evaluation obtained via the recommended controller besides various ones in Scenario No. 4.

Figure 31 depicts the$\:\:MOS\:$and $\:MUS$ values for frequencies and exchange tie-line power between dual areas. For example, the Fuzzy-PID controller has comparatively high overshoot values of 0.029534 Hz within area 1, 0.022255 Hz within area 2, and 0.003648 p.u. for tie-line power. The Fuzzy I-PD controller demonstrates improvements compared to the Fuzzy-PID controller by effectively dampening deviations to acceptable levels. Specifically, the Fuzzy I-PD controller maintains frequency deviations of 0.010551 Hz within area 1, 0.010577 Hz within area 2, and 0.001499 p.u. for power exchange. Moreover, the proposed controller achieves frequency deviations of 0.0061121 Hz within area 1, 0.0063521 Hz within area 2, and 0.001115 p.u. for tie-line power. It is evident that the recommended Fuzzy I-TD controller significantly reduces $\:MOS\:$and $\:MUS$ of $\:{\varDelta\:\text{f}}_{1\:}$by 79.30% and 94.76%, respectively, when compared to the Fuzzy-PID controller.

### Scenario no. 5: assessing the fuzzy I-TD controller in LFC at the two-area power grids considering ESSs types along with FACTS

#### Sub-scenario no. 5.1: studying the impact of different ESSs kinds

**Fig. 32:**
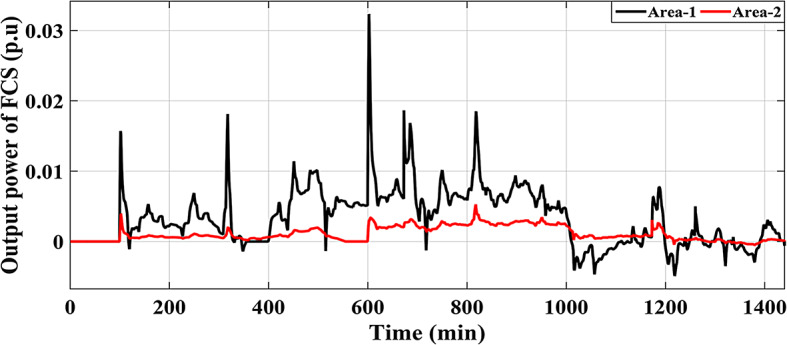
Controllable output power of the FCS in HRPGs for Scenario No. 5.1.

**Fig. 33:**
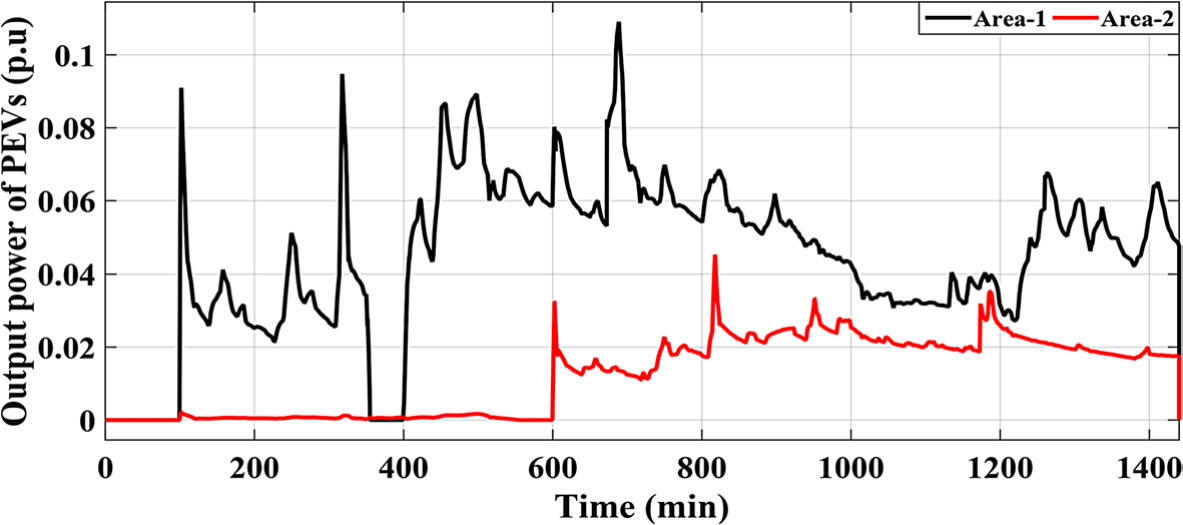
Controllable output power of the PEVs in HRPGs for Scenario No. 5.1.

**Fig. 34:**
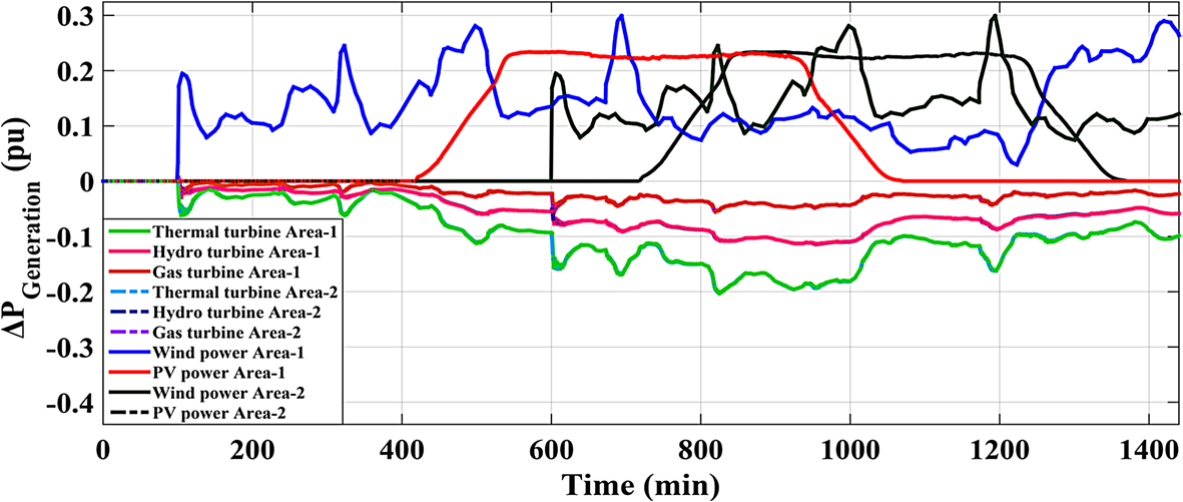
Output power of various RESs in HRPGs and the outputs of other generation sources for Scenario. 5.1.

**Fig. 35:**
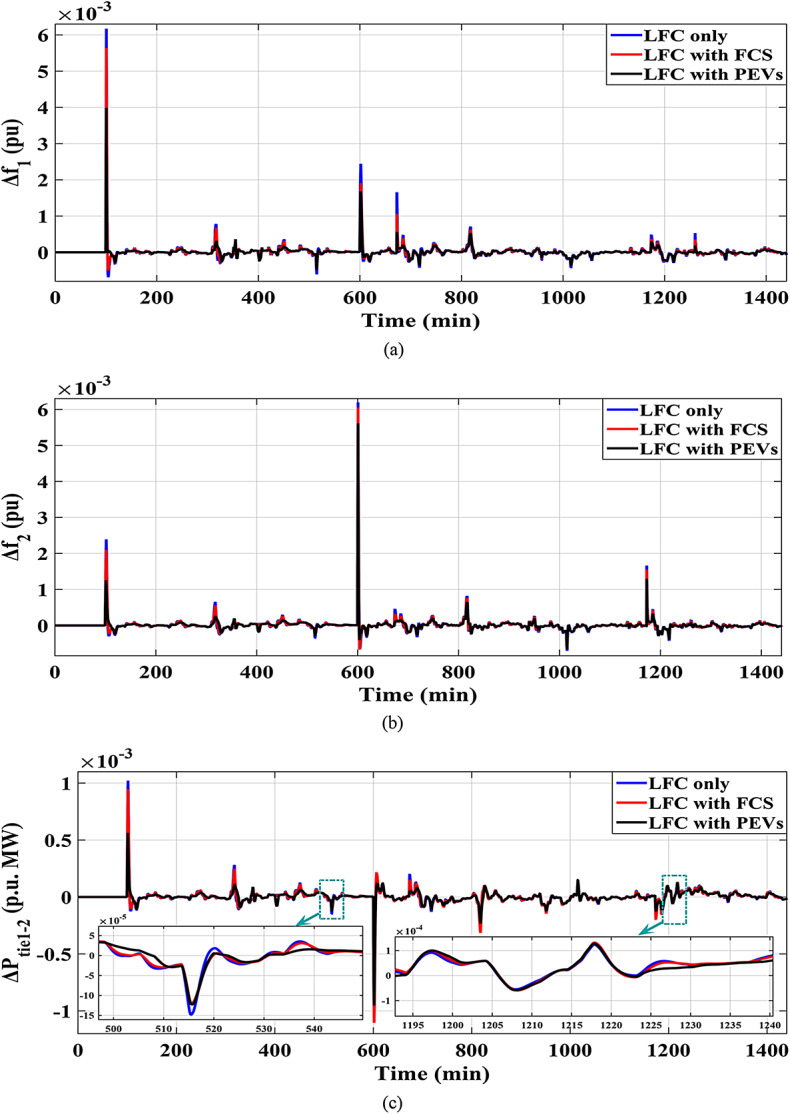
Results obtained in Scenario No. 5.1 (a)$\:\:{\varDelta\:\text{f}}_{1\:}$; (b)$\:{\:\varDelta\:\text{f}}_{2\:}$; (c)$\:\:{\varDelta\:\text{p}}_{\text{t}\text{i}\text{e}1-2\:}$.

This Sub-scenario discusses the influence of integrating several types of ESSs such as FCS and PEVs, as well as the effect of the recommended Fuzzy I-TD in LFC in reducing the analyzed HRPGs fluctuations. Whereas the controllable generated extra powers from FCS, PEVs and all considered generations in this grid are depicted in Figs. 32 and 33, and 34, respectively. The optimal PID controller parameters applied for controlling the output extra power are presented in Table 6. Additionally, Fig. 35 provides a comparative analysis of the dynamic performance of the examined HRPGs using the LFC only, the LFC with FCS, and the LFC with PEVs.

**Table 6:** The PID Controller’s optimal parameters relied on SHO algorithm for the proposed strategy for scenario no. 5.

Controller for ESSs	Area 1	Area 2
PID relied on SHO for PEVs	$$\:{k}_{p}$$ = 10$$\:,$$$$\:{k}_{i}$$ = 10$$\:,$$$$\:{k}_{d}$$ = 0.196	$$\:{k}_{p}$$ = 1.373$$\:,$$$$\:{k}_{i}$$ = 6.443$$\:,$$$$\:{k}_{d}$$ = 3.083
PID relied on SHO for FCS	$$\:{k}_{p}$$ = 9.941$$\:,$$$$\:{k}_{i}$$ = 9.989$$\:,$$$$\:{k}_{d}$$ = 0.184	$$\:{k}_{p}$$ = 1.385$$\:,$$$$\:{k}_{i}$$ = 6.502$$\:,$$$$\:{k}_{d}$$ = 3.0791

**Fig. 36:**
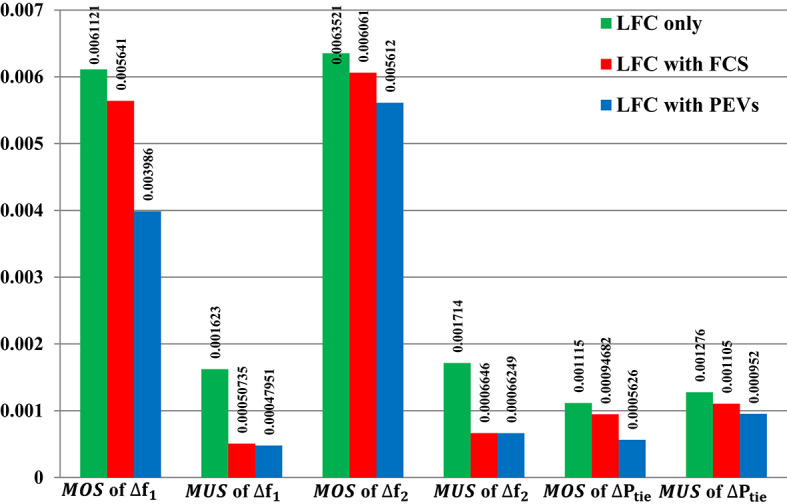
Performance evaluation obtained via the considered controller besides various ESSs in Scenario No. 5.1.

Figure 36 shows the$\:\:MOS\:$and $\:MUS$ values for frequencies and exchange tie-line power in dual areas. For instance, the recommended Fuzzy I-TD controller with PEVs has comparatively low undershoot values of 0.00047951 Hz within area 1, 0.0006131 Hz within area 2, and 0.00066249 p.u. for tie-line power. Following that, the second-best results are related to the proposed controller with FCS: 0.00050735 Hz within area 1, 0.0006646 Hz within area 2, and 0.001105 p.u. for tie-line power compared to utilizing the proposed LFC only. Moreover, the recommended Fuzzy I-TD controller with controlled PEVs significantly reduces $\:MOS\:$and $\:MUS$ of $\:{\varDelta\:\text{f}}_{1\:}$by 52.88% and 80.53%, respectively, when compared to the proposed controller in LFC only.

#### Sub-scenario no. 5.2: examining the impact of the proposed strategy

**Fig. 37:**
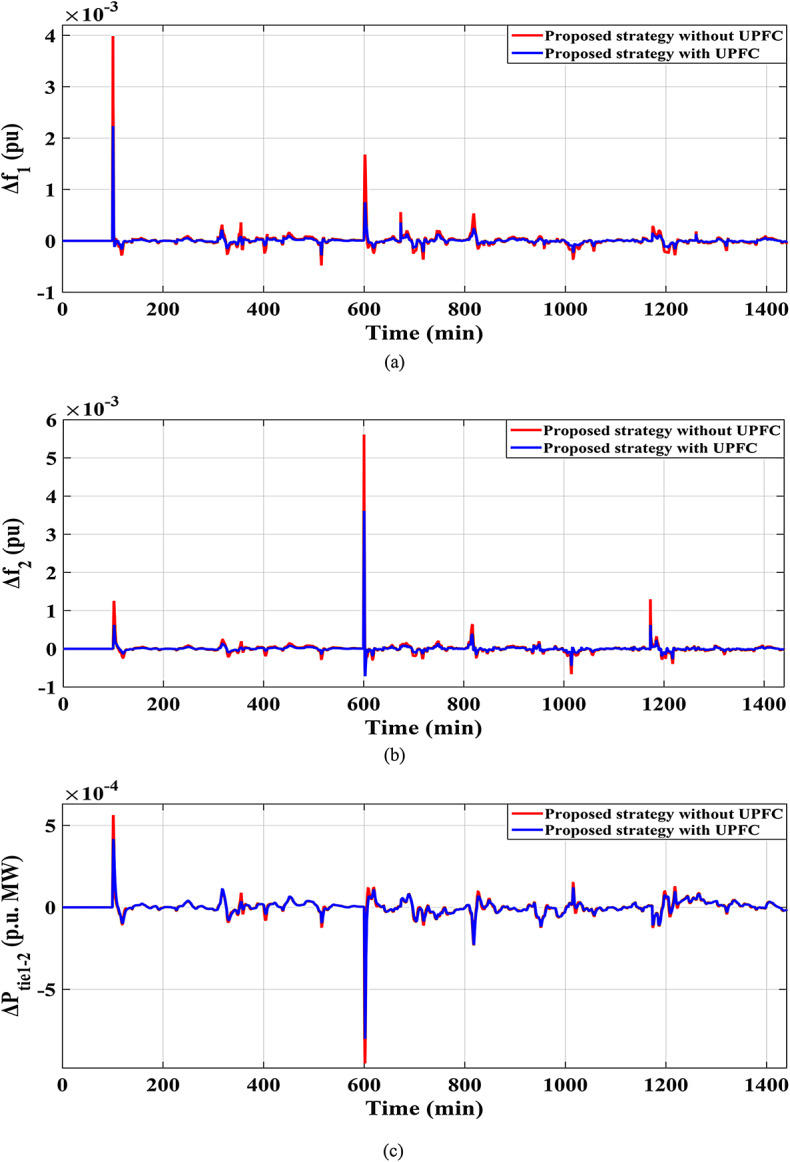
Results obtained considering the proposed strategy effect (a) $\:\:{\varDelta\:\text{f}}_{1\:}$; (b)$\:{\:\varDelta\:\text{f}}_{2\:}$; (c)$\:\:{\varDelta\:\text{p}}_{\text{t}\text{i}\text{e}1-2\:}$ for Scenario No. 5.2.

**Fig. 38:**
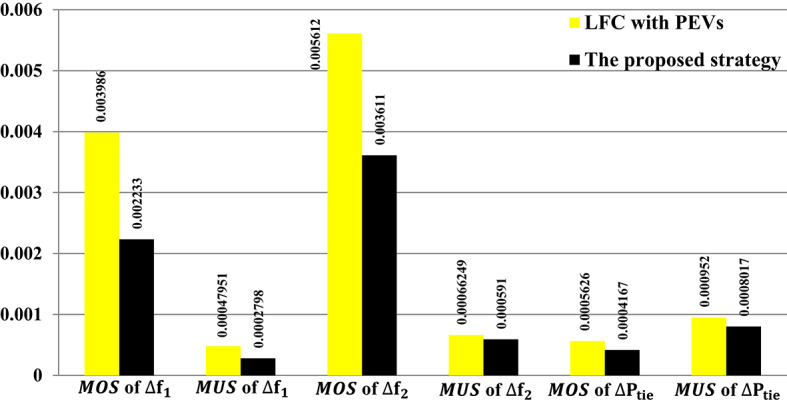
Performance evaluation obtained via the proposed strategy for Scenario No. 5.2.

Here, the effect of the impact of using FACTS such as UPFCs in dealing with the frequency stability problem has also been considered. The comparison has been conducted to the system with and without the UPFC installation considering the proposed strategy, is observed in Fig. 37, achieving more system dependability and stability. Additionally, Fig. 38 depicts the $\:MOS\:$and $\:MUS$ values for the interchange tie-line power and both-area frequencies among the dual studied areas. It can be observed that the recommended controller with the proposed strategy has comparatively low overshoot values of 0.002233 Hz within area 1, 0.003611 Hz within area 2, and 0.0004167 p.u. for tie-line power compared to results obtained utilizing the proposed controller with UPFCs. The inclusion of the proposed strategy with the UPFC in the investigated power grid results in a noticeable reduction in $\:MOS\:$and $\:MUS$ of $\:{\varDelta\:\text{f}}_{1\:}$by 22.47% and 11.46%, respectively, when compared to the proposed strategy without UPFC.

### Scenario no. 6: assessing the fuzzy I-TD controller in LFC in the IEEE 39-bus system

**Fig. 39:**
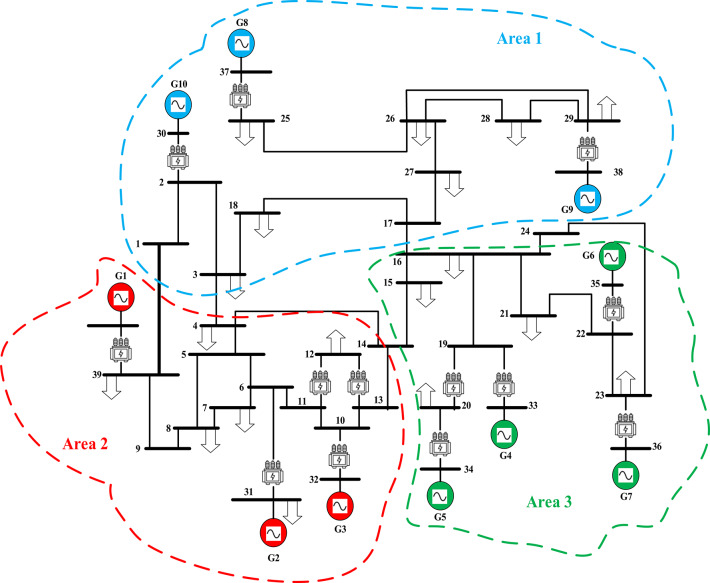
Schematic diagram of the IEEE 39-bus system^8,67^.

The recommended Fuzzy I-TD controller’s efficacy in LFC is evaluated utilizing the standard New England IEEE-39 bus system in this scenario. This system consists of ten reheat thermal generators, nineteen loads, thirty-eight transmission lines, and twelve transformers, as shown in Fig. 39. The system is organized into three interconnected local areas, namely local area 1, local area 2, and local area 3, with respective rated area power capacities of$\:{\:P}_{r1}=1500\:MW,{\:\:P}_{r2}=2000\:MW,$ and$\:{\:P}_{r3}=1500\:MW$. Table 7 lists the system parameter values. In this approach$\:,$ each area’s generating units are substituted with a single equivalent generation unit. The corresponding inertia constant and speed regulation characteristics can be computed using the formulas listed below^8,67^:

27 $$\:{H}_{eqv}=\frac{{H}_{1}{S}_{1}+{\:H}_{2}{S}_{2}+\dots\:+{H}_{n}{S}_{n}}{{S}_{system}}$$

28 $$\:{R}_{evq}=\frac{1}{\frac{1}{{R}_{1}}\left(\frac{{S}_{1}}{{S}_{system}}\right)+\frac{1}{{R}_{2}}\left(\frac{{S}_{2}}{{S}_{system}}\right)+\dots\:+\frac{1}{{R}_{n}}\left(\frac{{S}_{n}}{{S}_{system}}\right)}HZ/{MW}_{pu}$$

29 $$\:{S}_{system}={S}_{1}+{S}_{2}+\dots\:+{S}_{n}$$

where$\:{\:S}_{i},$ $\:{R}_{i}\:$and $\:{H}_{i}$ refer the specified generation unit’s power rating$\:,$ the factors of both speed regulation and inertia constant respectively related to $\:{i}^{th}$ generation units.

**Table 7:** Parameter values of IEEE 39 buses system as a representative model of a power system.

Local Area No.	Power generating unit	$\:\varvec{H}$	$\:\varvec{R}$	$\:\varvec{D}$	$\:{\varvec{T}}_{\varvec{g}}$	$\:{\varvec{T}}_{\varvec{t}}$
1	1 2 3	70.0 30.0 35.8	0.05 0.05 0.05	1 1 1	0.08 0.08 0.08	0.04 0.04 0.04
2	4 5 6 7	28.6 26 34.8 26.4	0.05 0.05 0.05 0.05	1 1 1 1	0.08 0.08 0.08 0.08	0.04 0.04 0.04 0.04
3	8 9 10	24.3 34.5 20.0	0.05 0.05 0.05	1 1 1	0.08 0.08 0.08	0.04 0.04 0.04

**Fig. 40:**
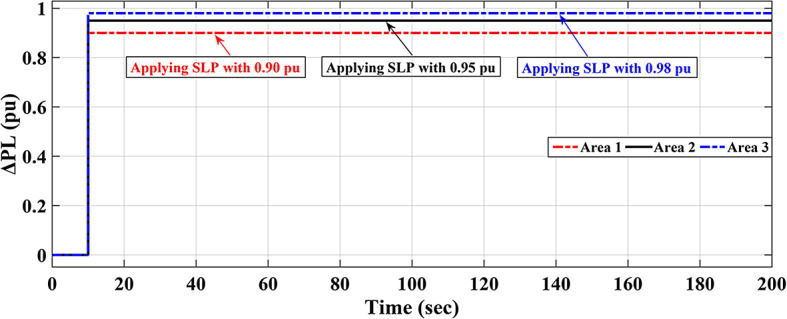
Different load disturbances applied in the three interconnected local areas.

**Fig. 41:**
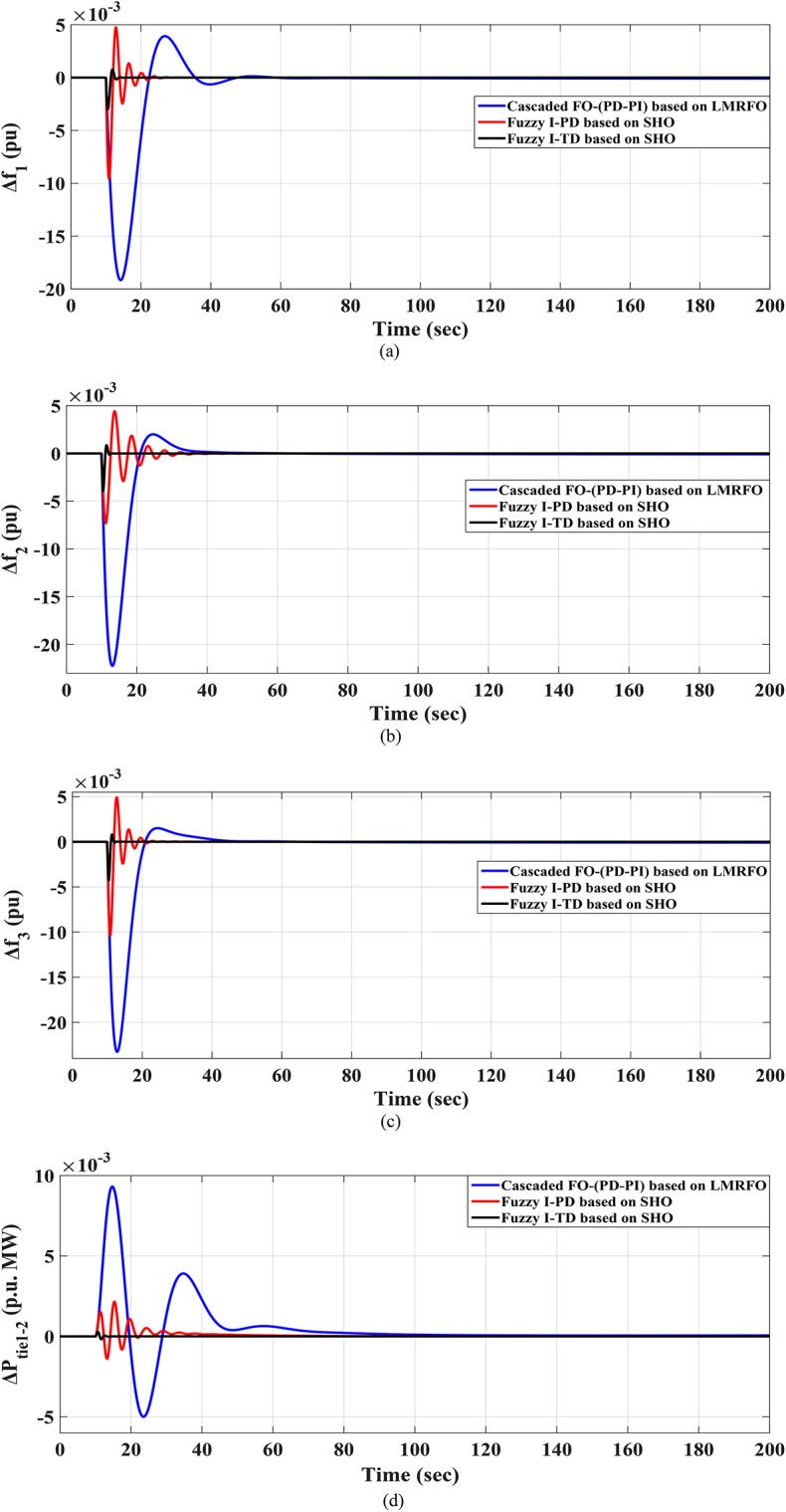

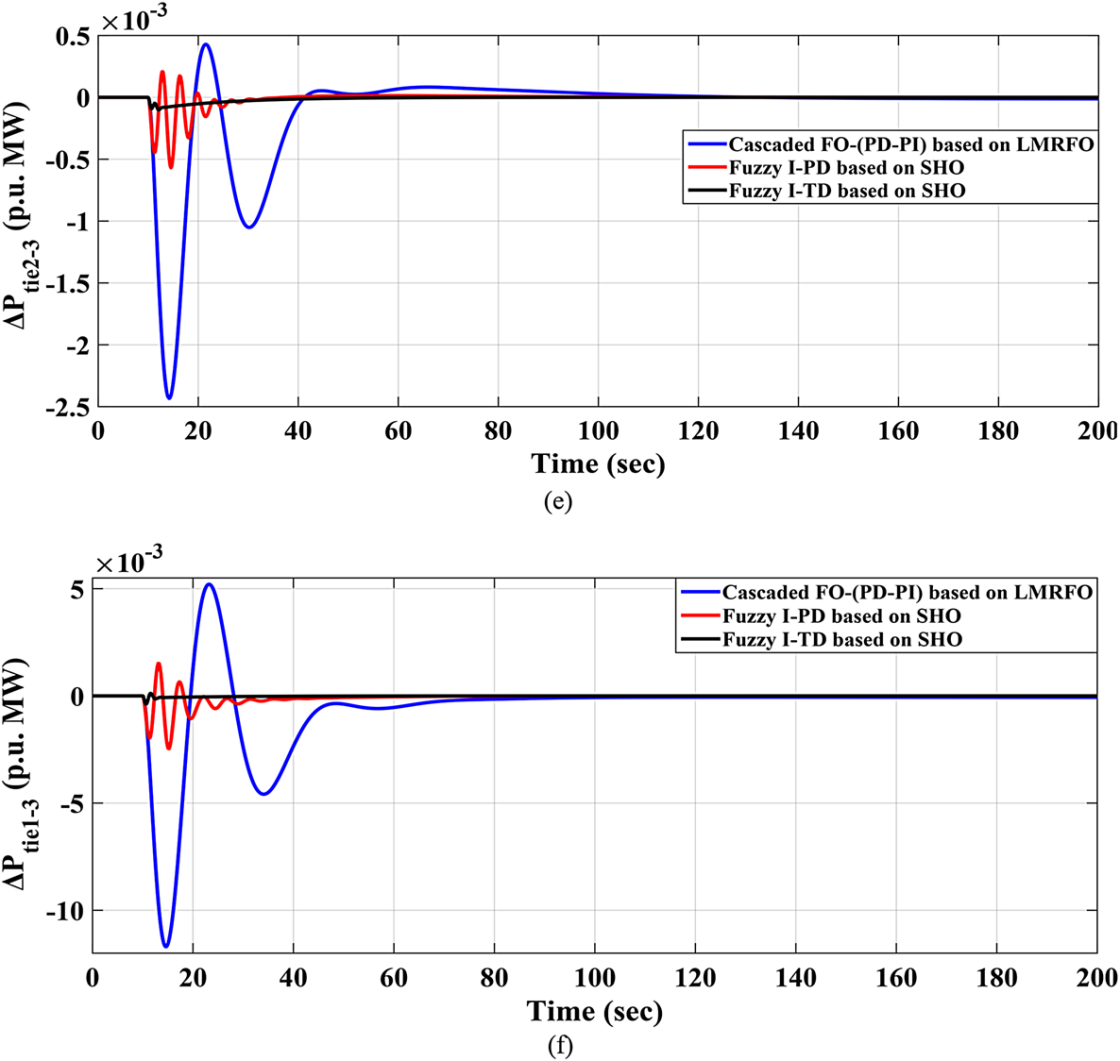
Results obtained in Scenario No. 6 (a)$\:\:{\varDelta\:\text{f}}_{1\:}$; (b)$\:{\:\varDelta\:\text{f}}_{2\:}$; (c)$\:{\:\varDelta\:\text{f}}_{3\:}$; (d)$\:\:{\varDelta\:\text{p}}_{\text{tie}1-2}$; (e) $\:{\varDelta\:\text{p}}_{\text{tie}2-3}$; (f) $\:{\varDelta\:\text{p}}_{\text{tie}1-3}$.

Furthermore, the optimal parameters of the proposed Fuzzy I-TD controller are listed in Table 8. Then, the frequencies of the three interconnected local areas and the power exchange in various tie lines demonstrate shorter settling times, decreased overshoots, and decreased undershoot. Specifically, the considered controller outperforms the cascaded FO-(PD-PI) controller used in^8^ and Fuzzy I-PD controller. Figure 40 displays the distinct SLD profiles experienced by the three interconnected local areas. The system’s dynamic responses are demonstrated in Fig. 41; highlight the recommended controller’s substantial efficacy in minimizing excursions and preserving system stability.

**Table 8:** The suggested Controller’s optimally optimized parameters for scenario no. 6.

Controller Description	Local Area 1	Local Area 2	Local Area 3
Fuzzy I-PD Based on SHO	$$\:{k}_{1}$$= 5.3709$$\:,$$$$\:{k}_{2}$$= 1.9129$$\:,$$$$\:{k}_{p}$$= 0.0894, $$\:{k}_{i}$$= −4.6988, $$\:{k}_{d}$$= −1.00008×$$\:{10}^{-6}$$	$$\:{k}_{1}$$= 5.3709$$\:,$$$$\:{k}_{2}$$= 1.9129$$\:,$$$$\:{k}_{p}$$= 0.0894, $$\:{k}_{i}$$= −4.6988, $$\:{k}_{d}$$= −1.00008×$$\:{10}^{-6}$$	$$\:{k}_{1}$$= 5.3709$$\:,$$$$\:{k}_{2}$$= 1.9129$$\:,$$$$\:{k}_{p}$$= 0.0894, $$\:{k}_{i}$$= −4.6988, $$\:{k}_{d}$$= −1.00008×$$\:{10}^{-6}$$
Fuzzy I-TD Based on SHO	$$\:{k}_{1}$$= 0.4736$$\:,$$$$\:{k}_{2}$$= 0.0925$$\:,$$$$\:{k}_{t}$$= −1.5614×$$\:{10}^{-11}$$, $$\:{k}_{i}$$= −10, $$\:{k}_{d}$$= −0.6728, *n* = 1	$$\:{k}_{1}$$= 0.4736$$\:,$$$$\:{k}_{2}$$= 0.0925$$\:,$$$$\:{k}_{t}$$= −1.5614×$$\:{10}^{-11}$$, $$\:{k}_{i}$$= −10, $$\:{k}_{d}$$= −0.6728, *n* = 1	$$\:{k}_{1}$$= 0.4736$$\:,$$$$\:{k}_{2}$$= 0.0925$$\:,$$$$\:{k}_{t}$$= −1.5614×$$\:{10}^{-11}$$, $$\:{k}_{i}$$= −10, $$\:{k}_{d}$$= −0.6728, *n* = 1

### Scenario 7: assessing the strategy (fuzzy I-TD with PEVs) in the modified IEEE 39-bus system

**Fig. 42:**
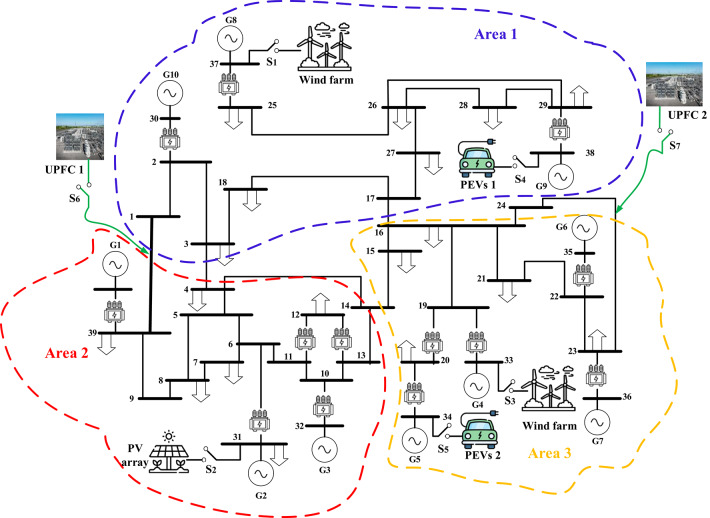
Schematic diagram of the IEEE system with renewables penetration.

**Fig. 43:**
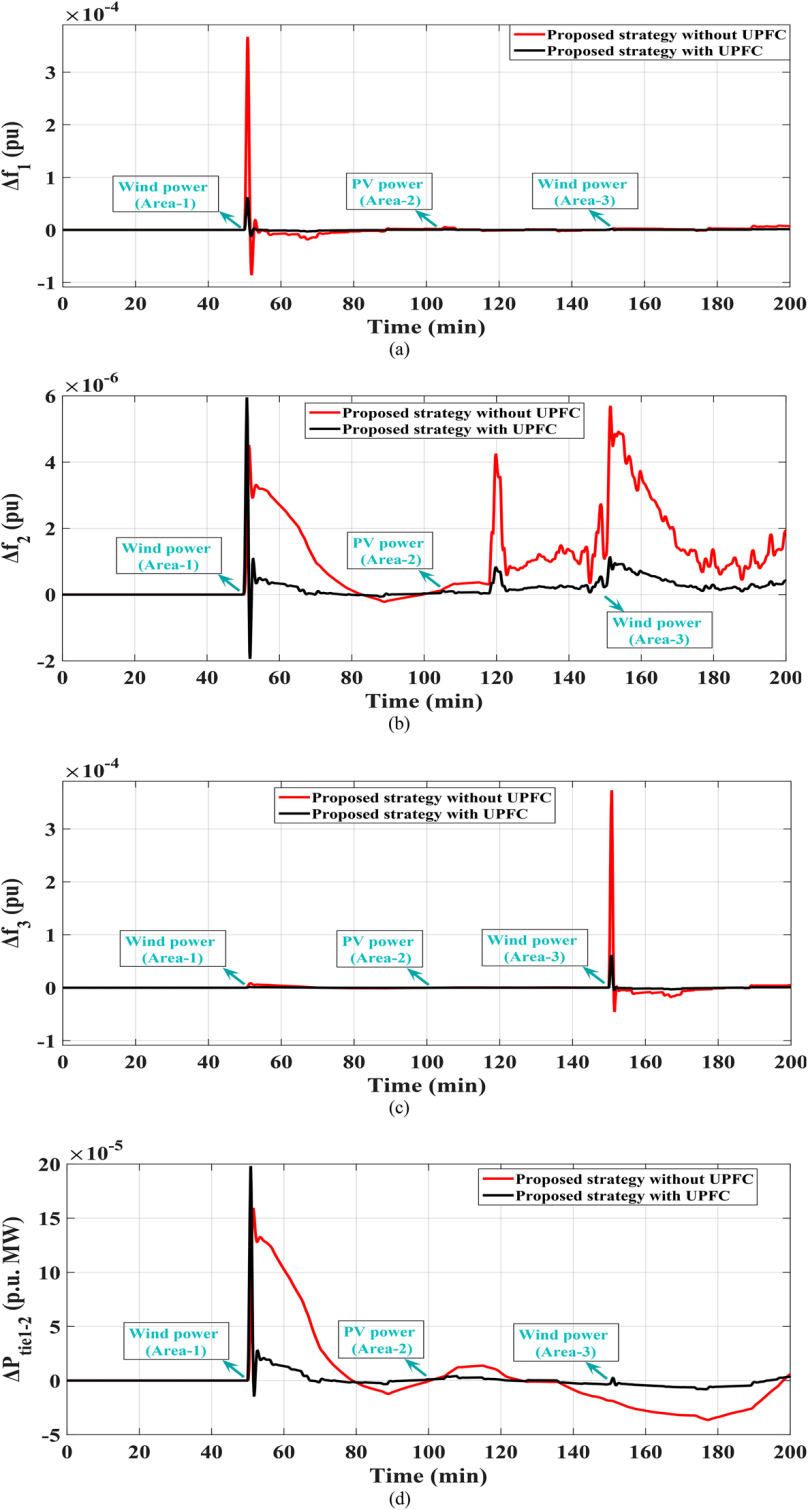

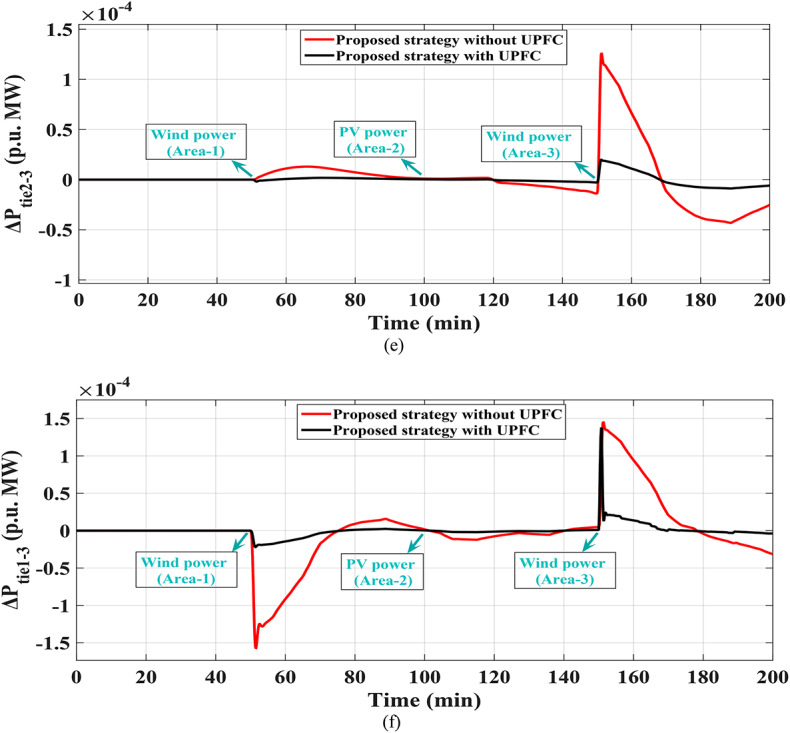
Results obtained in Scenario No. 7 (a)$\:\:{\varDelta\:\text{f}}_{1\:}$; (b)$\:{\:\varDelta\:\text{f}}_{2\:}$; (c)$\:{\:\varDelta\:\text{f}}_{3\:}$; (d)$\:\:{\varDelta\:\text{p}}_{\text{tie}1-2}$; (e) $\:{\varDelta\:\text{p}}_{\text{tie}2-3}$; (f) $\:{\varDelta\:\text{p}}_{\text{tie}1-3}$.

In this scenario renewables penetration has been considered in IEEE 39 buses standard system to evaluate the efficacy of the proposed strategy. Furthermore, the renewables penetration is considered as follows: Wind energy in area 1 at t = 50 min, PV energy in area 2 at t = 100 min, Wind energy in area 3 at t = 150 min. Each of PEVs and UPFC at t = 0 min; from the beginning of the simulation process. The standard IEEE system considering renewables is shown in Fig. 42. The system’s dynamic responses are presented in Fig. 43; highlights the proposed strategy substantial performance in minimizing excursions and preserving system stability.

## Conclusions

This manuscript has addressed the critical challenge of frequency deviations in hybrid renewable power grids (HRPGs) with high penetration of renewable energy sources (RESs). The study employed a Fuzzy I-TD controller, designed using a recent optimizer known as the Sea Horse Optimizer (SHO), to optimally tune the controller parameters for achieving superior performance in load frequency control (LFC) of HRPGs. The effectiveness of the presented methods has been validated through extensive simulations on two models: a two-area power grid and the IEEE 39-bus system. The key findings and efficacy of the studied strategies are summarized as follows:

- Optimization Effectiveness: The SHO demonstrated superior performance in optimizing controller parameters, outperforming traditional optimizers such as the Teaching Learning-Based Optimization (TLBO), Arithmetic Optimization Algorithm (AOA), and an Eagle Strategy Arithmetic Optimization Algorithm (ESAOA).
- Controller Design: In the secondary control loop (SCL), a distinctive Fuzzy I-TD controller has been implemented, outperforming conventional controllers such as Fuzzy-PID and Fuzzy I-PD. Under various operating conditions, the Fuzzy I-TD controller significantly reduced frequency and tie-line deviations by 82.7% and 97.01%, respectively, compared to Fuzzy I-PD and Fuzzy-PID. This highlights its superior capability in mitigating frequency instability in HRPGs.
- Integration of Energy Storage Systems (ESSs): The incorporation of controlled ESSs, such as plug-in electric vehicles (PEVs), further enhanced the system’s dynamic response. The Fuzzy I-TD controller with PEVs reduced frequency fluctuations by 40% compared to the Fuzzy I-TD controller alone, demonstrating the synergistic benefits of combining advanced control strategies with PEVs.

In conclusion, the proposed Fuzzy I-TD controller, combined with PEVs and optimized using the SHO algorithm, represents a significant step forward in addressing frequency stability challenges in HRPGs. The work may be extended through are several avenues for future research such as expanding the presented approaches to larger and more complicated power arrangements, involving multi-area grids with diverse RESs formations and exploring additional technological integrations to further advance the field.

## Data Availability

The datasets generated during and/or analyzed during the current study are available from the corresponding author on reasonable request.

## Appendix

See Tables 9, 10, 11, 12 and 13.

**Table 9:** The mathematical formulas for the HRPGs^26^.

Description of A Block	Formulation of Transfer Functions
HRPG_1_	$\:\frac{{K}_{psa}}{{T}_{psa}\bullet\:\text{s}+1}$
HRPG_2_	$\:\frac{{K}_{psb}}{{T}_{psb}\bullet\:\text{s}+1}$
Hydro Turbine	$\:\frac{-{T}_{w}\bullet\:\text{s}+1}{(0.5{).T}_{w}\bullet\:\text{s}+1}$
Transient Droop Compensation	$\:\frac{{T}_{rs}\bullet\:\text{s}+1}{{T}_{rh}\bullet\:\text{s}+1}$
Hydro Governor	$\:\frac{1}{{T}_{gh}\bullet\:\text{s}+1}$
Reheater of Thermal Turbine	$\:\frac{{K}_{r}\bullet\:{T}_{r}\bullet\:\text{s}+1}{{T}_{r}\bullet\:\text{s}+1}$
Thermal Governor	$\:\frac{1}{{T}_{sg}.\text{s}+1}$
Thermal Turbine	$\:\frac{1}{{T}_{t}.\text{s}+1}$
Gas turbine’s speed governor	$\:\frac{{x}_{c}\bullet\:\text{s}+1}{{Y}_{c}\bullet\:\text{s}+1}$
Gas Turbine Dynamics	$\:\frac{1}{{T}_{cd}\bullet\:\text{s}+1}$
Gas turbine’s valve positioner	$\:\frac{1}{{b}_{g}\bullet\:\text{s}+{c}_{g}}$
Fuel System and Combustor	$\:\frac{{T}_{cr}\bullet\:\text{s}+1}{{T}_{fc}\bullet\:\text{s}+1}$
Synchronizing coefficient	$\:\frac{{(2.pi.T}_{ab})}{\text{s}}$

**Table 10:** The values of HRPGs parameters^26^.

The Symbol	The Standard Value
$\:{B}_{i}$	0.4312 MW/Hz
$\:{\:T}_{12}$	0.0433 MW
$\:{R}_{1}$ $\:{R}_{2}$ $\:{R}_{3}$	2.4 HZ/MW 2.4 HZ/MW 2.4 HZ/MW
$\:{\:\text{a}}_{12}$	−1
$\:{K}_{T}$	0.543478
$\:{K}_{H}$	0.326084
$\:{K}_{G}$	0.130438
$\:{K}_{ps}$	68.9566
$\:{T}_{ps}$	11.49 s
$\:{T}_{sg}$	0.08 s
$\:{T}_{t}$	0.3 s
$\:{K}_{r}$	0.3
$\:{T}_{r}$	10 s
$\:{T}_{gh}$	0.2 s
$\:{T}_{rs}$	5 s
$\:{T}_{rh}$	28.75 s
$\:{T}_{w}$	1 s
$\:{b}_{g}$	0.05
$\:{c}_{g}$	1
$\:{Y}_{c}$	1 s
$\:{X}_{c}$	0.6 s
$\:{T}_{cr}$	0.01 s
$\:{T}_{fc}$	0.23 s
$\:{T}_{cd}$	0.2 s
$\:{K}_{EV}$	1
$\:{T}_{EV}$	0.28 s
$\:{K}_{FC}$	0.03
$\:{T}_{FC}$	3 s

**Table 11:** The parameters related to the wind power plants^26,54^.

Parameters	Definitions
$\:{A}_{T}$	Swept sectional area ($\:{m}^{2})$
$\:{V}_{W}$	Speed of wind ($\:m$/$\:s$),
*ρ*	Density of air ($\:kg$/$\:{m}^{3}$),
$\:\lambda\:$	Tip-speed ratio (TSR)
$\:{C}_{p}$	Rotor blades coefficient
*β*	Blade pitch angle
$\:{\omega\:}_{B}$	Blade’s angular velocity (rad/sec).

**Table 12:** The parameters related to the PV plants^8^.

Parameters	Definitions
$\:{\eta\:}_{sc}$	cell reference’s efficiency
$\:{\alpha\:}_{sc}$	solar cell absorbance
$\:{\tau\:}_{g}$	transmissivity of glass
$\:R$	the solar radiation (W/$\:{m}^{2}$),
$\:{\mu\:}_{sc}$	the thermal coefficient of PV cell efficiency ($\frac{\% }{{^{ \circ } {\text{C}}}}$)
$\:{T}_{sc}$	temperature of the solar cell (${^{ \circ } {\text{C}}}$)
$\:{T}_{r}$	The reference temperature (${^{ \circ } {\text{C}}}$).

**Table 13:** The solar panel cell temperatures under various sunlight circumstances^8^.

Radiation (W/$\:{\mathbf{m}}^{2}$)	Solar cell temperature (${^{ \circ } {\text{C}}}$)	
582.13	36.78	
595.18	37.38	
626.32	38.3	
668.56	39.22	
714.235	40.175	
760.48	41.025	
790.78	42.23	
815.61	44.615	
820.4725	46.395	
840.4825	47.12	
841.4075	47.655	
